# Designing Asynchronous Remote Support for Behavioral Activation in Teenagers With Depression: Formative Study

**DOI:** 10.2196/20969

**Published:** 2021-07-13

**Authors:** Arpita Bhattacharya, Ria Nagar, Jessica Jenness, Sean A Munson, Julie A Kientz

**Affiliations:** 1 Department of Informatics University of California, Irvine Irvine, CA United States; 2 Psychological Science University of Glasgow Glasgow United Kingdom; 3 Department of Psychiatry and Behavioral Sciences University of Washington Seattle, WA United States; 4 Human Centered Design and Engineering University of Washington Seattle, WA United States

**Keywords:** teens, mental health, behavioral activation, asynchronous remote communities

## Abstract

**Background:**

Many teenagers in the United States experience challenges with symptoms of depression, and they lack adequate resources for accessing in-person mental health care. Involving teens and clinicians in designing technologies that use evidence-based practices that reduce barriers to accessing mental health care is crucial. Interventions based on behavioral activation (BA) help teens understand the relationship between mood and activity, help them practice goal-directed behaviors to improve mood, and may be particularly well-suited to delivery via internet-based platforms.

**Objective:**

This study aims to understand the needs and challenges that teens and mental health clinicians face in depression management and involve them in the design process of a remote intervention that uses asynchronous remote communities. Our goal is to understand the benefits and challenges of adapting BA to an internet-based platform that supports the asynchronous remote community approach as a delivery tool for teen depression management.

**Methods:**

We enrolled mental health clinicians (n=10) and teens (n=8) in separate, private, internet-based groups on Slack (Slack Technologies Inc). They participated in 20-minute design activities for 10 weeks and were then invited to interviews about their experiences in the study.

**Results:**

Both teen and clinician participants wanted internet-based support for BA as a supplement to in-person therapy. Although participants perceived the asynchronous format as conducive to supporting accessible care, teens and clinicians raised concerns about safety, privacy, and the moderating of the internet-based group. Design decisions that address these concerns need to be balanced with the potential benefits of learning coping skills, increasing access to mental health care, and promoting asynchronous human connection to support teens.

**Conclusions:**

We discuss considerations for balancing tensions in privacy and safety while designing and selecting internet-based platforms to support remote care and integrating evidence-based support when designing digital technologies for the treatment of teens with depression.

## Introduction

### Background

Approximately 3.2 million teenagers are diagnosed with depression each year [1]. Depression in teens is a serious and debilitating disorder associated with lifelong negative outcomes, including increased risk of recurrence into adulthood, social difficulties, physical illness, and suicidality [1-5]. More than 60% of adolescents with depression do not receive in-person mental health care, and among those who do, treatment engagement is low [1,6]. Evidence-based psychosocial interventions (EBPIs) for individuals with depression typically require frequent interactions between patients and mental health providers throughout time, which can be a barrier for patients and costly to administer in person [1].

Technology-based tools may provide an opportunity to improve the usability and engagement of EBPIs, particularly among teens where daily technology use is nearly ubiquitous [7]. Asynchronous remote communities (ARCs) are technology-mediated groups that use private internet-based platforms to deliver weekly tasks to participants in a format that is lightweight, accessible, usable, and low burden [8]. ARCs capitalize on the reach of technology while also providing support, social interactions, and motivation to engage in care. In this study, we seek to engage teens and mental health clinicians in the design process to understand how to use an ARC platform to support an EBPI for depression.

### Overview of Behavioral Activation Therapy

**Figure 1:**
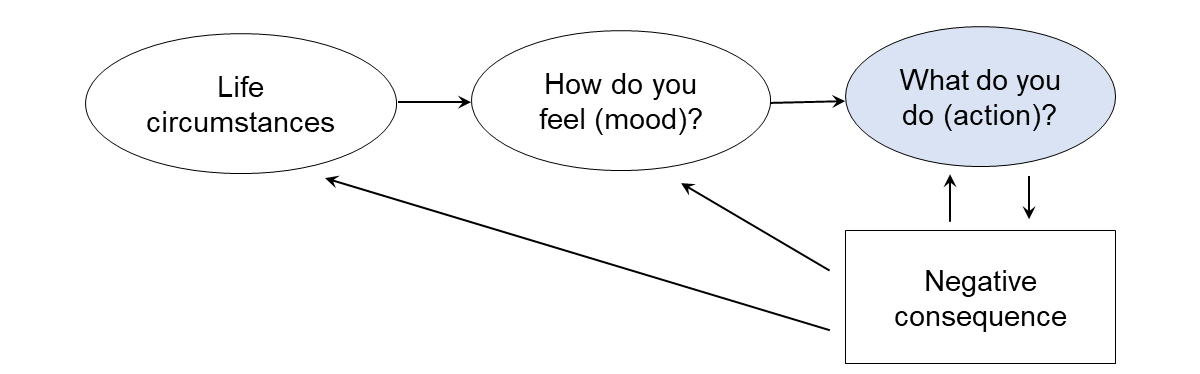
Behavioral activation model of depression among teens.

Behavioral activation (BA) is an EBPI for individuals with depression [9-11] and lends itself to a wide range of implementation and training methods [12,13]. BA is based on a functional analytic model of depression that highlights the transactional associations among environmental stress, behavior, and mood (Figure 1) [10]. Specifically, BA approaches the treatment of depression through two primary targets: (1) increasing the experience of positive reinforcement (rewarding experiences) to help improve mood and (2) decreasing avoidance of reinforcing activities that may negatively reinforce depression symptoms. To address these treatment targets, BA emphasizes practicing goal-directed rather than mood-directed behavior. For example, if a teen is rejected by a friend and subsequently experiences a low mood, a mood-directed behavior would be to isolate from all peers to avoid further rejection. Isolation from a broader group of peers would likely lead to negative consequences, such as worsening friendships and a lack of social contact. These negative consequences feedback in the teen’s environmental stress, low mood, and avoidant behaviors, ultimately resulting in a negative cycle of depression. Alternatively, goal-directed behavior refers to setting and following small steps toward a goal (instead of a mood) that aligns with the teen’s core values and is likely to positively influence their mood. For example, despite the mood-directed inclination to avoid potential rejection, the teen may set a goal to see a movie with a friend and follow small steps toward this goal, such as calling the friend, setting a time and date, and selecting a movie to watch. Although BA holds promise as an effective treatment for depression [11,14], there is an opportunity to improve the usability of and engagement with BA via internet-based technologies, particularly among teens who are highly engaged with social and mobile technologies [7,15,16].

### Designing for Teen Mental Health

Technology can be creatively designed to engage teens in EPBIs for emotional and mental health management. Researchers have used different modalities of engagement for preteens and teens, such as engaging parents and children with strategies for social and emotional skill learning through digital storytelling and dialogic inquiry [17] and designing a toy that provides real-time biofeedback to mitigate negative emotional responses [18]. Researchers have also designed a social robot (Ecological Momentary Assessment Robot) [19] with teens to assess their stress and support emotive and embodied interactions. In a pilot randomized controlled trial of a website delivering BA modules on planning activities to improve mood, Davidson et al [12] found that 96.2% of teen participants who had depression symptoms completed the module. They suggested tracking activities and moods and sending reminders using mobile technology to improve engagement [12]. Rohani et al [20] designed an app called MORIBUS based on BA for activity planning and rating and visualizing mood patterns for adults with depression. In their feasibility study, participants found personal visual insights into the relationship between their mood and activity most useful with an overall compliance rate of 71%. Researchers identified opportunities to support the need for flexibility in logging activities instead of only in-the-moment logging, as participants had individual usage patterns. For example, some participants logged their activities at the end of the day when they had privacy, versus a few who logged it as they completed the activity. Flexibility in how and when individuals engage with internet-based mental health tools can be supported by an asynchronous format of logging and participating in activities.

### The Need for an ARC Method

When working with teens, researchers have encountered challenges in using common design methods (eg, curt or not fully formed responses, power imbalance, and access constraints), suggesting a need for new and innovative ways to involve them in design [21]. ARCs are private internet-based groups in which researchers can deliver periodic research tasks to participants and gather information about their perceptions in a format that is lightweight, accessible, usable, and low burden. Researchers have used the ARC method to engage adults living with chronic and stigmatized illnesses and people who face challenges with access to care [8,22,23]. Asynchronous methods have also been used in conjunction with internet-based social networking tools to engage with youth in intervention research [24,25]. For example, researchers enrolled 79 young adults in private Facebook groups to deliver cognitive behavioral therapy interventions for smoking cessation for 90 days [25]. SharpTalk was an internet-based, moderated peer support discussion forum designed for youths aged 16-25 years who engage in self-harm [24]. For teens, ARCs on social networking platforms offer more convenient and lightweight access than visiting offline research sites, as teens may encounter barriers to in-person appointments, such as the need for transportation and parental support. ARCs also support engaging with and following teens’ activities throughout time and teens who are geographically distributed. Our previous work highlighted ARCs as a promising approach for engaging teens in EBPIs for mental health that leverages technology’s reach while understanding teens’ challenges with mental health, needs for support and social interactions, and motivations [26]. In this study, we sought to expand the use of ARCs to understand the benefits and challenges of adapting BA to an internet-based platform that supports intervention delivery and engagement tools for teen depression management.

### Study Overview

In this study, we aim to use and adapt the ARC method to involve teenagers and clinicians in the design process of adapting BA interventions. We built our group guidelines and protocols for moderating the group and handling internet-based disclosures of adverse events based on ethical considerations on balancing teens’ safety, preference to remain anonymous, and potential distress by Sharkey et al [24] (Multimedia Appendices 1-3).

The following research aims guided the design of our internet-based activities, interviews, and analysis:

- Aim 1 (A1): using the ARC to understand perceptions, needs, and challenges of clinicians and teens in designing technologies for the treatment of teen depression
- Aim 2 (A2): understanding what clinicians and teenagers envision from the design and delivery of BA for the treatment of teen depression using internet-based technology platforms that support an ARC approach

To address these aims, we aim to use the ARC method with clinicians and teens in two separate, private, internet-based groups and post 20-minute–long design activities each week for 10 weeks. Each activity prompted clinicians and teens to provide feedback on adapting the BA to Slack (Slack Technologies Inc) and design implementation ideas. We seek to discover their needs, design constraints, and facilitators of and barriers to adapting BA for asynchronous delivery and analyzed their experiences with the ARC. This empirical work provides design recommendations for researchers and practitioners working with internet-based technologies to use EBPIs for teen depression management. On the basis of our analysis, both clinicians and teens highlighted (1) the need for technology to be a supplement to therapy and not a replacement, preserving the in-person interaction that therapy usually provides and (2) the importance of balancing human connection on the web while considering both privacy and safety.

## Methods

### Overview

We conducted two separate ARC studies on Slack with 10 mental health clinicians (C1-C10, including therapists, primary care, and school counselors), who worked with depressed teens, and 8 teen participants (T1-T8) aged between 15 and 19 years who experienced mild-to-moderate symptoms of depression. We aimed to understand clinicians’ and teens’ current perceptions, strategies, challenges, and technology use in managing depression (A1) and their design ideas for adapting BA to internet-based platforms that support an ARC approach to intervention (A2). We created separate groups for teens and clinicians to prevent power dynamics between teens and clinicians from influencing each other’s answers, creating separate spaces where each group could prioritize their own needs and preferences; thus, each group could communicate comfortably. We started the teen groups 3 weeks after the clinician group so that we could make changes to the activities based on clinicians’ feedback and use the teens’ time efficiently. Clinicians were recruited through snowball sampling of a network of researchers. To recruit teens, we contacted participants from an earlier study [26] who agreed to be recontacted, posted flyers in clinician participants’ clinics with their permission, and used snowball sampling. The study was approved by the Human Subjects Division of the University of Washington.

### Participants

In total, 10 clinician participants started the study, with 8 completing all internet-based activities. All clinicians (n=10) were from urban or suburban areas in Seattle and Kent, Washington (Table 1), and 8 had previous experience using BA with teens. We started the study with 8 teenage participants, and 4 teenagers dropped out by week 9. All teen participants identified as female (Table 2) and were from rural, suburban, and urban regions across the United States. A clinician dropped out in week 4 (because of a family emergency) and another dropped out in week 7 (because of difficulties with using the Slack platform; Table 3). The client base and setting of the clinicians included teens and preteens with depression (11-18 years), including school counseling, community clinics, pediatrics, primary care, immigrants, and refugees. The client base and setting of the clinicians included teens and preteens with depression (11-18 years), including school counseling, community clinics, pediatrics, primary care, immigrants, and refugees.

**Table 1:** Summary of demographic details of clinician participants (n=10).

Demographics			Values
Age (years), mean (range)			39 (31-50)
**Gender, n (%)**			
	Women	7 (70)	
	Men	3 (30)	
	Nonbinary	0 (0)	
**Education level, n (%)**			
	Graduate education	7 (70)	
	Professional degree	3 (30)	
**Race or ethnicity, n (%)**			
	White	8 (80)	
	Asian	1 (10)	
	Hispanic	1 (10)	
**Household income (US $), n (%)**			
	50,000-75,000	4 (40)	
	100,000-150,000	2 (20)	
	150,000-200,000	1 (10)	
	≥200,000	3 (30)	
**Region, n (%)**			
	Urban	8 (80)	
	Suburban	2 (20)	
	Washington	10 (100)	
**Experience with evidence-based practice, n (%)**			
	Cognitive behavioral therapy	8 (80)	
	Behavioral activation	8 (80)	
	Mindfulness-based approaches	6 (60)	
	Dialectical behavior therapy	4 (40)	
	Interpersonal psychotherapy	3 (30)	
	Acceptance and commitment therapy	2 (20)	
	Other	3 (30)	

**Table 2:** Summary of demographic details of teen participants in study 2 (n=8).

Demographics		Values
Age (years), mean (range)		17.5 (15-19)
**Gender, n (%)**		
	Women	8 (100)
	Men	0 (0)
	Nonbinary	0 (0)
**Education level, n (%)**		
	College education with degree	1 (13)
	Some college education but no degree	2 (25)
	High school	2 (25)
	Less than high school	3 (38)
**Race or ethnicity, n (%)**		
	White	5 (63)
	Asian	1 (13)
	More than one race	1 (13)
	Did not disclose	1 (13)
**Household income (US $), n (%)**		
	35,000-49,000	1 (13)
	75,000-100,000	1 (13)
	150,000-199,000	1 (13)
	≥200,000	2 (25)
	Did not disclose	3 (38)
**Region level, n (%)**		
	Rural	1 (13)
	Suburban	2 (25)
	Urban	5 (63)
	Washington	5 (63)
	Philadelphia	1 (13)
	Iowa	1 (13)
	New York	1 (13)
**Therapy experience, n (%)**		
	Received treatment for depression in the past	6 (75)
	In treatment during the study	5 (63)

**Table 3:** Summary of activities on the private Slack group.

Week	Design activity	Purpose and activities	Clinician completion rate (n=10), n (%)	Teen completion rate (n=8), n (%)
1	Introductions	Learning how to use features of Slack Getting to know other participants	10 (100)	7 (88)
2	Technology to manage mood	Sharing current strategies and technologies that participants have found helpful for their clients (clinicians) and themselves (teens and clinicians)	10 (100)	7 (88)
3	Internet-based mental health support, part 1	Learning about more features of Slack such as polls with example poll Ideating on benefits and challenges of using the internet-based platform of Slack	10 (100)	7 (88)
4	Internet-based mental health support, part 2	Voting polls on preferred features of Slack for internet-based support with mood Voting polls for the format of the internet-based support (eg, group vs individual) and the length of internet-based support	9 (90)	8 (100)
5	Adapting BA^a^ to the web	Psychoeducational video explaining BA Prototype: obtain feedback on a summary of BA activities for 6-week format (Multimedia Appendix 4)	9 (90)	7 (88)
6	BA Model and activity monitoring	Explaining BA model with a video and concepts through slides (Figure 2) and ask participants to give examples from their lives by uploading hand-drawings (Figure 3) Prototype: mock-up of mood and activity monitoring in survey format	8 (80)	6 (75)
7	Upward and downward spiraling of mood; introducing SMART^b^ goals	Providing feedback on upward and downward spiraling of mood and planning SMART goals Prototypes: Survey format for reflecting on upward and downward spirals in mood and action (Figure 4) Survey format for individually planning a SMART goal, mini-steps, and setting reminders	8 (80)	6 (75)
8	SMART goal planning	Providing feedback on technological adaptations of planning a SMART goal, mini-steps, and setting reminders Prototypes: Mock-up of chatbot format Direct messaging format in which participants pair up with a peer and a researcher moderator. Participants were asked to share SMART goals and provide feedback on each other’s goals	7 (70)	6 (75)
9	Overcoming barriers	Providing feedback on overcoming barriers to mini-steps and SMART goals. Prototypes: Survey format Chatbot format with prompts to overcome barriers (Figure 5) Direct messaging format by pairing up with a peer from week 8, following up, and sharing barriers	7 (70)	4 (50)
10	Teaching components	Identifying how to deliver teaching components of BA in internet-based formats such as videos and chatbots	7 (70)	4 (50)
11-12	Exit interviews and surveys	Providing feedback on the method and follow-up questions on depression management	9 (90)	5 (63)

^a^BA: behavioral activation.

^b^SMART: specific, measurable, appealing, realistic, and timebound.

**Figure 2:**
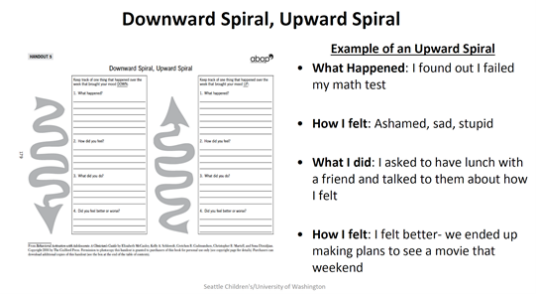
Example of a slide (PDF) of instructions presented to teens to explain the Downward and Upward Spiral Worksheet for behavioral activation.

**Figure 3:**
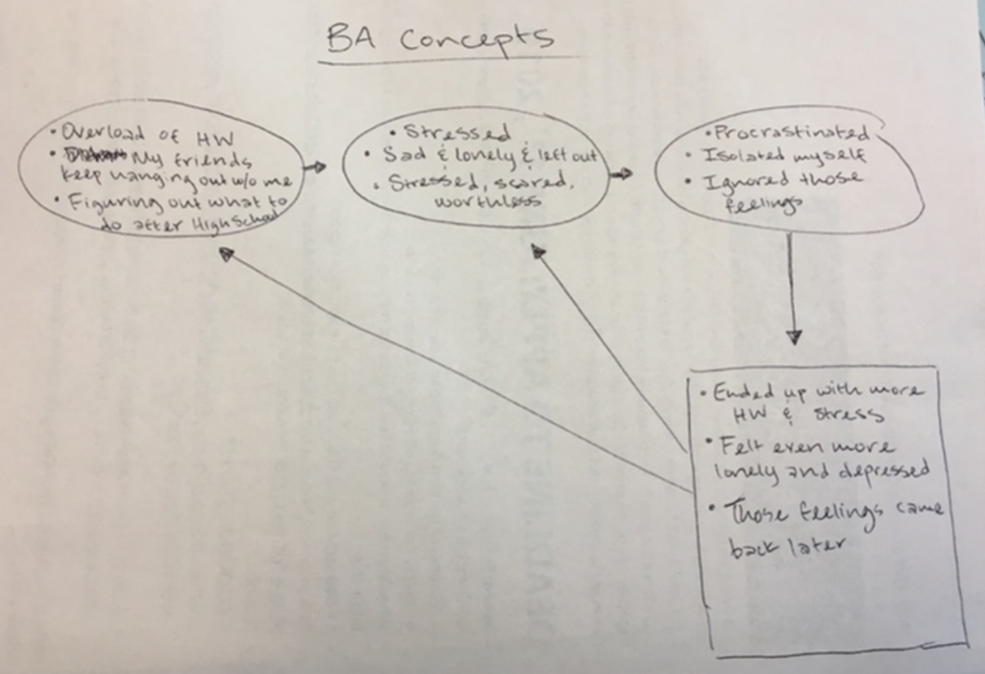
“How behavioral activation (BA) works for me?” Hand-drawn picture from a teen participant explaining how BA would apply to their life circumstances. BA: behavioral activation.

**Figure 4:**
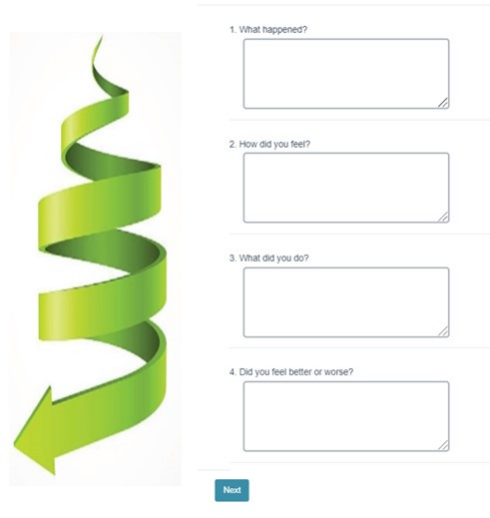
Mock-up for the internet survey–based adaptation of the downward spiral module in behavioral activation.

**Figure 5:**
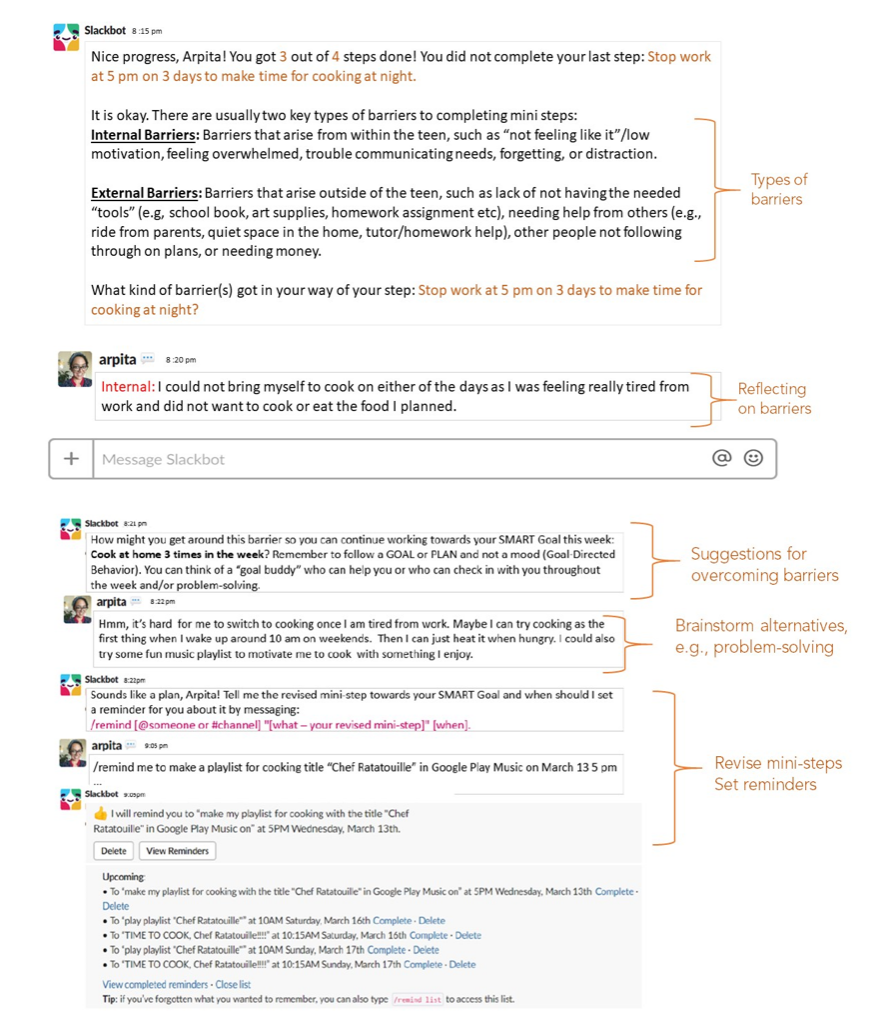
Teen and clinician preferences for features of internet-based support. The figure contains the first author’s name as an example to protect participants’ privacy.

### Survey Measures

At the beginning of the study, teens completed the Patient Health Questionnaire-8 (PHQ-8) adolescent scale [27]. Although some teens did not identify with having experienced clinical levels of depression (n=1), all teen participants had mood ratings on the PHQ-8 in the 5 (mild depression) to 14 (moderate depression) range (mean 8.87, SD 3.14). In the poststudy surveys, we asked all teen and clinician participants to complete the acceptability, intervention appropriateness, and feasibility of intervention measures [28] and user burden scale [29] surveys to determine participant approval of and burden using BA delivered on an internet-based platform.

### Internet-Based Group Activities

We conducted 10 weekly activities in the clinician group followed by 10 weekly activities in the teen groups (Table 3; Multimedia Appendix 4), each designed to take 20 minutes to complete. The groups were moderated, and all clinicians and teen participants selected a pseudonym to use as their username on Slack to protect their privacy.

### Poststudy Interviews

To learn about clinician and teen participants’ experiences with depression treatment, the ARC method, and further explore ideas for implementing BA via an ARC platform, we conducted 30- to 40-minute exit interviews with 9 clinicians and 5 teens and surveys with participants between June and August 2019. All interviews were audio-recorded and transcribed professionally. The study protocol is provided in Multimedia Appendix 5.

### Design of Prototypes of BA Activities

We created low-fidelity prototypes to show how some BA activities might be adapted to an interactive, asynchronous remote platform and showed them to our clinician and teen participants to obtain feedback. Prototypes included low-fidelity paper-based or survey-based mock-ups and screenshots, and the research team could easily incorporate feedback and iterate on the design before investing resources into building the tool (eg, Figures 2-5). For each module in weeks 6 to 10, we posted worksheets from the BA handbook [1] for take-home activities and low-fidelity prototypes of the technological adaptations of these take-home worksheets (Table 3). On the basis of feedback from clinicians, we presented each BA module to the teen groups by using examples on 2-3 slides (Figure 2) to briefly introduce and explain each activity along with the technology mock-ups of the survey or chatbots. All participants were asked to try and review adaptations in the form of surveys, voting polls, group and direct messages on Slack; upload photographs; and critique chatbot mock-ups. We asked participants to provide feedback on the content and format of these activities and their engagement and experiences with these activities.

In week 9, we posted a module to overcome barriers to SMART (specific, measurable, appealing, realistic, and timebound) goals that they planned in week 8. In the first part of this activity, participants were given examples of barriers and suggestions to overcome these barriers. These mock-ups also included survey prompts, a chatbot mock-up, and direct messaging with peers (Figure 4). In week 10, we presented mock-ups of possible teaching formats when delivering modules of BA. These remote teaching formats included animated videos to explain BA modules, teen peers explaining based on their lived experiences, and an interactive format using a chatbot where the respondent can use dialog and voting polls.

### Analysis

We calculated the average scores of the PHQ-8 [27] and each question of the acceptability, intervention appropriateness, and feasibility of intervention measures [28] for the clinician group and teen groups, respectively. For the user burden scale [29], we computed the average scores of teens and clinician groups separately across each of the 6 constructs: physical, mental and emotional, time and social, financial, difficulty of use, and privacy. By “making and analyzing thematic connections” [30], 2 researchers inductively analyzed the qualitative data each week from the clinician and teen groups and the interview transcripts by developing codes. Both coders first independently coded a subset of the data corresponding to the same weeks and discussed codes together to prepare a codebook (Multimedia Appendix 6). Coders then separately coded all the data based on the codebook, reviewed the codes together, discussed and resolved any discrepancies in coding, and wrote memos. We discussed the results with the entire team and used our research aims to guide an affinity diagramming process [31], through which our final two themes emerged.

### Ethical Considerations

Important considerations for using ARC with minors include maintaining privacy and confidentiality, ethical handling of adverse event disclosures on the web (such as suicidality, abuse, or harassment), and the possibility of distress for others in a group setting. We obtained the emergency contact information of an adult from all teen participants and informed the teens that this person would be contacted if they disclosed medical emergencies or concerns of harm to self or another. With the consent forms, we asked the participants to review group guidelines and pinned them on the Slack group. We explicitly stated on the consent form, group guidelines (Multimedia Appendix 1), and the first day of activities that we were not professional counselors but were willing to listen to grievances and provide them with 24-7 helpline numbers such as Teen Link and the National Suicide Prevention helplines to reach out to professionals. We had protocols for internet-based disclosures of adverse events (Multimedia Appendix 2) and child abuse (Multimedia Appendix 3) in place for the research team. Teens were informed that they could expect a response within 1 business day if they contacted the moderator with questions or concerns. Both the clinician and teen groups were monitored by a moderator who could contact a licensed psychologist with doctoral-level training in child clinical psychology (Jessica Jenness, PhD) if any safety or emergency concerns arose. Moderators read all the posts, monitored for safety concerns and emotional distress, and reached via email or Slack private message in case of concern. No immediate risks of physical harm or abuse were disclosed during the study (Multimedia Appendices 1-3).

## Results

### Overview

At the beginning of the study, teens and clinicians were asked about their preference for a short (4-6 weeks) or long format (12 weeks) of BA. Most teens and clinicians voted for the short format, so we tailored our design activities to a short BA format. We observed that teen participants started dropping out around week 5. The 5 teens who completed 9-10 activities and were interviewed at the end of the study said they wanted the long format to familiarize themselves with, learn, and practice BA strategies. In the following sections, we explain three themes that emerged from our analysis of the needs of teens and clinicians for internet-based support in managing mood and depression (A1): (1) balancing the need to augment human connections and asynchronous BA support and (2) the need for boundaries around asynchronous internet-based support.

### Balancing Needs for Asynchronous BA Support and Augmenting Human Connection

When presented with the idea of including an automated chatbot application on the internet-based platform, both teen and clinician participants perceived the chatbot’s role as an interactive platform for learning, self-reflection, supplementing resources when therapists are not available, and supporting treatment planning. Interactive asynchronous activities included engaging with psychoeducational videos**,** tracking mood and activity, self-reflection, planning, and check-ins.

#### Interactive Learning

We presented mock-ups of internet-based surveys and chatbots as self-help technological adaptations of homework activities for BA. Clinicians expressed the need to include interactive, culturally, and generationally relevant features (such as images in the graphics interchange format and emoticons) to increase engagement with teens. Both teen and clinician participants appreciated the use of chatbots and their potential for responsiveness and interactivity, step-by-step guidance, availability at all times, ability to store and post lists of relevant information, and added explanations and reinforced motivation. Clinicians also perceived that teens would find it engaging and “be into it” [C8, clinician]. Clinicians brainstormed how a chatbot could provide additional information to support teens that clinicians might miss or not have time to discuss during short appointments.

#### Support Self-reflection

Teens found the private internet-based survey format to be simple, clear, and helpful in illustrating BA concepts by applying written examples from their own context. They explained that the prompts in the activity on upward and downward spiraling of mood (*what happened, how did you feel, what did you do,* and *how did that make you feel*) helped structure their thoughts and helped them reflect. They also found it to be a good balance between journaling both positive and negative effects of an action on mood. Two teens also found it helpful that the BA prompts forced them to write about and focus on a specific situation:

I think sometimes I can get overwhelmed with emotion in situations, and it really helps to take a breath and think, what triggered me? How did I feel? And how can I avoid feeling this way in the future? Writing out “I felt sad, mad and overwhelmed” really helps me process the emotions I felt!

T4

Clinicians highlighted that there should be some way for teens to record how they were feeling or their mood at a specific time and then get prompted to do something that could possibly *help them in-the-moment* to alleviate symptoms.

#### Planning Support

Planning and executing SMART goals are a crucial aspect of the BA. To learn about SMART goals and have an option where teens could interact with other teens, a moderator created four small chat groups with herself and 2 randomly paired teen peers on direct message. We asked each teen in the pair to share a SMART goal, provide feedback on the other’s goal, share mini-steps to attain that goal, and set reminders for mini-steps on Slack or their phones; 2 teen pairs completed this activity:

Thanks! My smart goal is to write five thank you cards to teachers by the end of next week.

T2

Hi @T2! My smart goal is to clean up my room over the weekend. I think your goal is Very smart! It’s specific (writing cards), measurable (5 cards), appealing, realistic and timebound (by the end of the week), good job :two_hearts:

T4

That sounds good! And thanks so much!! I like ur goal but my one question is how will you know when you are done/how can you measure your progress?

T2

Ooo that’s a good question actually...I’ll know I’m done when the entryway is cleared and everything in that area is sorted and put back!

T4

Sweet! Very smart :))

T2

In the other two groups, a teen posted their SMART goals, and the other teen did not respond. When asked to post about barriers to attaining that goal in the next week’s activity on direct message, only one pair of teens completed the activity. Although we sought to foster connection and accountability, such a lack of interaction can be counterproductive for this vulnerable population. During interviews, teens speculated that this problem might be addressed if we could add more activities in the beginning for teens to get to know each other. When asked if they would prefer smaller groups, teens preferred having 4-5 peers in each group so as not to be overwhelming and still enough social capital if some were not participating. During interviews, a teen explained that the SMART goals she and her partner selected were trivial, and it would be more beneficial if they could select more appropriate goals that would benefit their mood. As they could not tell if the partner accomplished the goal other than through self-report, she said it did not help hold herself accountable. Another teen explained that they liked to work independently. Therefore, it is important to preserve a space where teens can practice and learn on their own. A clinician suggested adding digital rewards in the self-help mode if they indicated completing the mini-steps:

I like the chatbot mockup -- I would have it programmed so that with each mini step completion they get some kind-of visual reward like fireworks or a giphy if they complete all of their steps. Even if they do not, there could be some kind of visual message like you got this!

C5

Clinicians also thought that they might want to offload reminders to a chatbot, automating the process of reminding teens to complete assigned materials or activities.

#### Augmenting Human Connection

Most teen and clinician participants did not prefer an entirely remote-only format and wanted to increase access to human-human support for mental health. They critiqued the chatbot format, perceiving that it could be impersonal and increase isolation compared with formats that develop human connections. In addition, 3 teens mentioned that chatbots could get repetitive; thus, it would be easy to ignore notifications:

Being in the States I often feel isolated since people value independence and not bothering others. Yet, the way to make relationships is actually through asking for help. If the robot [chatbot] or on-line discussions are made available, it seems to be reinforcing the isolation.

C6

**Figure 6:**
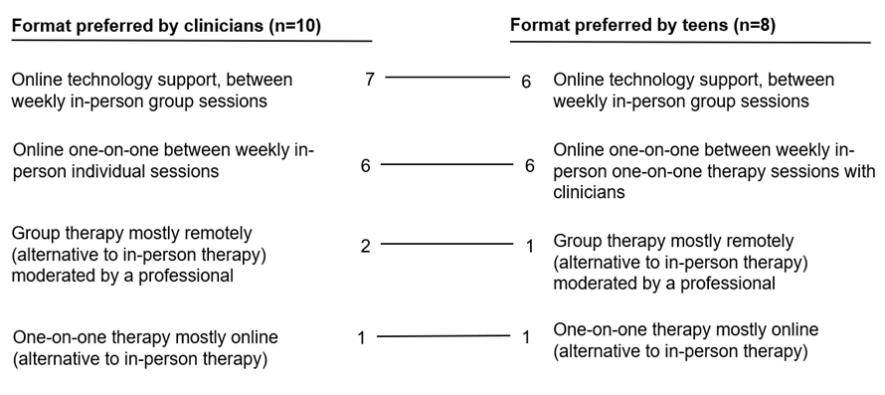
Preferences for the format of the internet-based delivery of behavioral activation (individuals could select more than 1). The values represent the number of teens and clinicians who voted for the respective formats. Trend lines connecting them illustrate the similarities in preferences of clinicians and teens.

**Figure 7:**
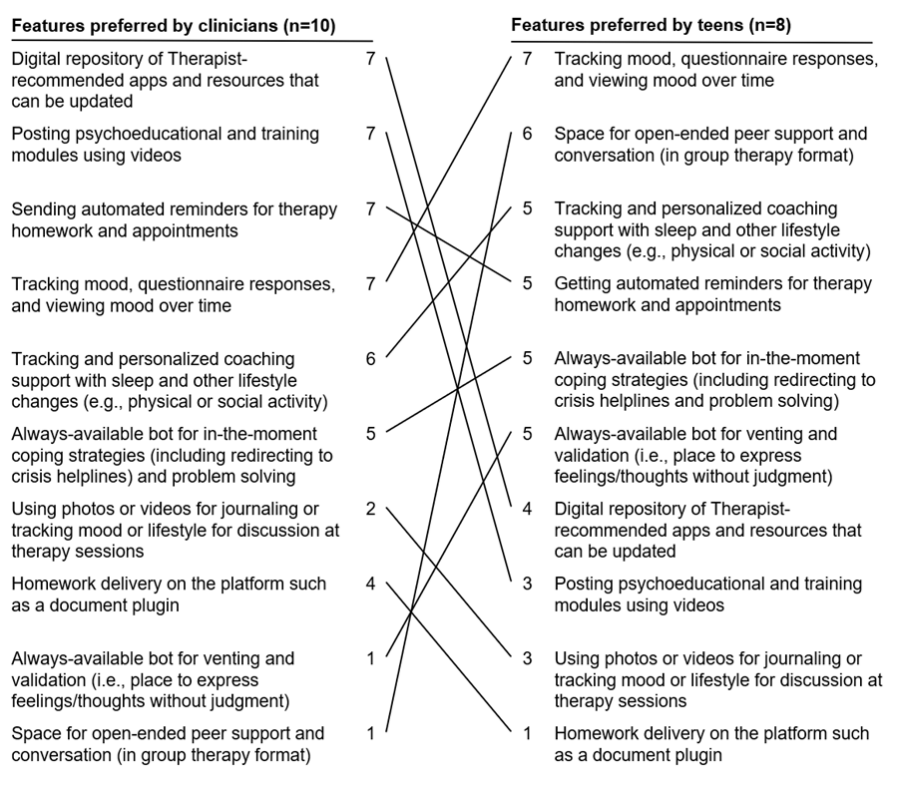
Teen and clinician preferences for features of internet-based support. The values represent the number of teens and clinicians who voted for the respective features. The lines facilitate the comparison of the rankings of preferences among clinicians and teens.

In total, 5 clinicians and 6 teens wanted one-on-one internet-based support in addition to in-person therapy (Figure 6 and Figure 7). Teens explained that they wanted to preserve the face-to-face therapy format but felt the burden of time, transportation, cost, and frequency of weekly therapy visits. They said they would prefer internet-based video chats or phone calls to connect with therapists rather than replacing in-person therapy entirely with asynchronous chat:

I think my challenges would be feeling lonely with a lack of human connection (especially in talking to a chat bot). I would be willing to try it, but I believe I might begin to feel isolated if the only thing that I can talk to/will listen is a programmed robot.

T5

A total of 6 teens preferred a group support format on the web in addition to one-on-one therapy. At the end of the study, teens mentioned that learning strategies from each other and building off each other’s ideas were helpful for the Slack group. When asked what they were looking for in peer support, they elaborated—empathy, a platform to share struggles and be *heard* by a human, benefits in the ability to express themselves in writing or talking out loud, and relating to shared experiences of a peer who is going through similar difficulties:

Social media shows the highlights and the best moments and it’s hard to remember that nobody’s life is perfect. Having a platform where you can discuss your problems and give advice is refreshing.

T6

Clinicians also highlighted the need for an internet-based platform where their teen clients can interact with peers (who have similar struggles and cultural backgrounds) who may be difficult to access offline. A school counselor, who worked with teens that were primarily immigrants and not fluent in English, requested different languages to deliver the treatment and connect the minority population with similar peers on the web:

For example, if I’m seeing an Arabic speaking kid, if there is an Arabic speaking group online, I think it would be really helpful to have an additional group where he can connect with other people in their own native language. There’s psychoeducation, there’s a little learning, there’s encouragement from his peers. I think it’s great, because I think in a way it probably will help connect with people, he’s not able to connect with in our school because there is not enough people in counseling, or open to counseling who also speak Arabic.

C6

Thus, participants emphasized the need to augment human interactions by increasing therapist-teen interactions and peer interactions using an internet-based platform.

### Need for Boundaries Around Asynchronous Internet-Based Support

The concerns of clinicians and teens using Slack during our study centered on the need for boundaries around privacy, safety during a crisis, and time burden on clinicians because of asynchronous access. They anticipated these concerns when using an internet-based platform for delivering mental health therapy.

#### Privacy

In our study, teens wanted to remain anonymous in the group and did not want to share identifying information such as real names, email addresses, or phone numbers. We asked all participants at the start of this study to deidentify (such as removing names and blur pictures) or not share any identifiable information about themselves and to not share information about others outside the group, even if they knew the participant in person. During the interviews, all teens expressed that these guidelines helped reduce their concerns regarding sharing in the group. However, 2 teens expressed concerns about their data being shared with people who were not a part of the group. These invisible audiences included a parent potentially finding and reading information on the group and the web company’s policy around sharing data with a third party:

Some parents might be more intrusive—or I feel like that could be a problem for some people who might be concerned that their parents would go and—if it was text-based, go see what they’ve been saying and stuff like that.

T1

Oh my God, yes, a thousand [privacy] concerns. I think that is really hard to trust online services to not sell your data. And I’m with therapy, sometimes it’s super confidential. So I think that it’s different from person to person—my concerns would be about tech companies sharing my data.

T2

In contrast to this preference for *external* privacy, some teenagers felt a lack of reciprocal interactions between peers *within* the group. Teens attributed this to the inability to know other teens in the group on a *personal level*, that is, sharing their interests and values while remaining anonymous. Although it was attributed to the lack of personal connection, Slack not being a part of the teens’ regular social media use could have also contributed to this less frequent interaction.

Although participants took measures to remain anonymous, Slack did not offer end-to-end encryption or Health Insurance Portability and Accountability Act (HIPAA) protection for personal health data shared on the platform. Clinicians have brought this up as a major issue. They explained that if such a platform were to be adopted by their clinical institution in the future, it had to be HIPAA compliant.

#### Safety

The second major concern discussed by both teen and clinician participants was the physical and emotional safety of teens on the internet-based platform. As the discussion format is asynchronous, the current internet-based group is *available* or accessible by teens at all times. Clinician participants were concerned about unnoticed posts at nonworking hours that need urgent attention, such as indicating suicidality or self-harm. They were also concerned about secondary exposure to distress and sharing and learning unhealthy or maladaptive coping behaviors in a peer group format:

Issues around teens messaging a therapist when a therapist isn’t available to respond—like if the teen posts at 2am that they are feeling suicidal and nobody sees it until the next day.

C3

I would be concerned about how teens’ interactions would be monitored/shaped in an anonymous group – I would want to think more about safety concerns (e.g., suicidality) and how to communicate about this in a timely, safe, and not overly reactive manner.

C4

Some clinicians wanted the chatbot to be programmed to identify words related to crises such as suicidality and alert a human who can help or provide a list of resources to the teen.

Teens brought up similar concerns of being triggered by others’ difficult experiences and not being able to share or minimize their struggles if they felt their experience was not as difficult as someone else’s in the group:

The problems may be that hearing about others’ problems more regularly thanks to the openness and limitless-ness of a chat format could have an effect on one’s own mental health, especially if the chat ends up just being a place to rant and only has negative thoughts filling it up, instead of any productive or supportive conversation.

T1

One challenge/problem is that people who have mood disorders/depression might exacerbate someone else’s hardships if they’re having a bad time. For instance, if John is really depressed and talks about his problems at home, Joe might not feel like he can talk about his own problems because they aren’t as “bad” as John’s.

T3

Clinicians felt a need to always be available on the web for safety reasons, which would be prohibitive for their workload. They explained the concerns and needs for setting boundaries and expectations around receiving and responding to crisis messages and reviewing homework submitted on the web:

I would also be concerned that clients would be reliant on immediate responses from me. As they do with most social media. Would have to coach them on their expectations.

C2

Clinicians expressed concerns about how they would be compensated for the time spent on the web and reviewing homework. Some clinicians believed that they would have difficulty teaching content on an internet-based platform in an effective manner. They also mentioned that more monitoring or moderating of the group might take more time, possibly with an extra cost. Clinicians acknowledged the potential benefits of the internet-based platform for delivering treatment via an ARC format, and they wanted the treatment to be as effective with less added burden on them:

A challenge that hasn’t been discussed here is billing. I know it’s a little unsavory to bring up, but if there are features of this system that require monitoring by a professional, then this seems like non-billable time, which in our current healthcare system is difficult to find.

C1

Overall, participants indicated a low burden and high adaptability of the internet-based intervention. The poststudy survey was completed by 9 clinicians and 5 teenagers. On the user burden scale (1: not at all burdensome; 4: very burdensome) [29], participants scored an average of 1.36 (clinicians) and 0.7 (teens) on *difficulty of using Slack*, 0.4 (clinicians) and 0.5 (teens) on *mental and emotional burden,* 0.4 (both) on *privacy burden*, and 0-0.1 on all other types of burden. The interview data also reflected that the teens found no or less difficulty using Slack compared with clinicians. A teen expressed privacy concerns about how Slack shared her data. Other teens had no concerns about privacy, and all felt comfortable sharing in the group when anonymous. The clinicians talked about issues with privacy related to HIPAA, but their score on the privacy burden was low. The average scores on the acceptability, intervention appropriateness, and feasibility of intervention measures of the intervention (1: not at all; 5: very much) [28] averaged between 3.5 and 4.8, indicating high perceptions of adaptability.

## Discussion

### Summary of Results

The key findings of this study included the need for (1) augmenting human connection in therapy and (2) establishing boundaries around asynchronous communication for the safety and privacy of participants. Teens and clinicians both preferred the use of an internet-based platform for psychoeducation, homework activities, check-ins, reminders, and self-reflection between one-on-one therapy sessions. Both groups preferred that it not be a technology-only intervention and wanted the platform to connect teens with a therapist or peers.

### Chatbots and Other Interactive Tools in Augmenting Mental Health Therapies

Clinicians recommended increasing the *human-like* conversation style of the chatbot to connect with and engage teens. However, teens did not want a chatbot to replace or emulate a human but envisioned its function as an interactive tool that scaffolded self-reflection. This is a promising approach. Research in other settings has shown that such people may prefer chatbots to questionnaires and that they may lead to greater engagement and more reliable and higher quality responses than questionnaires [32]. Conversational agents can also support reflection and self-learning in the workplace [33] or around physical activity goals [34].

However, their use is not challenging. Chatbots can fail to handle or escalate errors in the wild; people can perceive bots’ human-like responses as irrelevant or eerie (also known as *the uncanny valley*), and not everyone prefers to interact with a system that emulates human behaviors [35-37].

Researchers and designers must also consider what expectations might be set by a human-like agent and whether the chatbot is up to meet those expectations before using it to offload or augment human labor. As personal safety of disclosure of suicidal content was a major concern for clinicians, they considered a task-focused bot [35] would be appropriate to flag crisis posts 24-7 and escalate it to a human clinical expert. However, any such bot would need to be extensively tested before being relied upon for such a role. It would need to be regularly re-evaluated to ensure it was staying ahead of any changes in the use of language throughout time. Such an evaluation would need to be robust across different demographic groups, with particular attention to historically marginalized groups, to mitigate bias in existing data sets. Both teens and clinicians preferred chatbots in the role of intelligent assistants [35] to send and receive reminders and check-ins. Some teens expressed concerns about potential exposure to their peers’ distress, but neither teens nor clinicians wanted a chatbot to fulfill the roles of a virtual companion [35].

### Considerations for Platforms to Deliver Technology Support in Interventions

Previous research has identified the feasibility of using internet-based platforms to support BA treatment with teens [12] alongside the need for mobile platforms to support varying usage times and patterns of tracking BA activity [20]. We addressed these needs by obtaining formative feedback from teens and clinicians on the design of an asynchronous, modular, and weekly approach to delivering multiple BA intervention components while enabling access and flexibility for teens in tracking, planning, and reflecting on their activities and mood in situ. On the basis of the lessons from this study, we reflected on the aspects of the internet-based platform, procedures used by researchers, and the format of integrating internet-based interventions with traditional therapy that needs to be considered when designing future internet-based mental health interventions.

Reflecting on our study procedures and the use of an internet-based platform that supports an ARC approach, changes to the structure and facilitation of the internet-based group may help balance the need for human connection with safety in a peer group format. These changes include limiting the time of access to the group, an always-visible and easy-to-reach helpline button, distributed moderation, good moderation policies and communication of those policies, and/or automated in-the-moment crisis support. When deciding on a platform for ARC to deliver mental health interventions, we list the requirements for consideration by administrators or moderators (Textbox 1). Although Slack was a helpful tool, researchers might consider alternatives such as Microsoft Teams (HIPAA compliant but not anonymous), Discord (supports anonymity) [38], Group Me, or a custom-built internet-based platform, which can still allow the option to be anonymous on the group while being intuitive, familiar to teens, and able to organize and present content. During the COVID-19 pandemic, the ARC method is a safe and accessible method for human-computer interaction and clinical research.

**Important requirements from internet-based platforms and moderators or admins for asynchronous remote mental health interventions.**

**Support and limitations of internet-based platforms**

- Access
  Users should be able to access the platform on both computers and mobile phones to be able to use it in their context (eg, at school, at home, or between work). Having both a browser option and an app option helped participants who did not want to install anything.
  There is no additional cost for installation.
- Privacy: anonymity
  Users should be able to use pseudonyms when signing up.
  Administrators should have the option to hide emails and other personal identifiers on the internet-based platform.
- Privacy: health care
  Trying to attain Health Insurance Portability and Accountability Act (HIPAA) compliance would be the gold standard.
  If HIPAA compliance is not possible, make sure that teens are anonymous, are not interacting with clinicians, and are in separate groups in a study.
  Consider the scalability of using the platform in the real world with clinics
- Safety
  Internet-based platforms should allow pinned posts with 24-hour helpline numbers for crisis support to be accessible at all times.
  Participants should be able to access moderators via direct messaging. Need clear affordances for group participants to contact moderators and helplines
  Set expectations about moderator hours and response times (eg, expect a response within 24 hours on weekdays)
  Have access to clinician researchers or clinical support (eg, partnering with a local clinic) on a group and have adverse events protocols in place for crisis response
  Moderators can be supported with Chatbots to help them filter adverse events and alert them on urgent issues.
- Group norms
  The internet-based platform should have affordances such as pinned posts or sidebars with *always-accessible* group expectations, guidelines, and norms.
- Creating apps and bots
  Internet-based platforms should have a public application programming interface (API) to create and add bots and apps.
- Exporting data
  API of the internet-based platform should allow exporting data
- Content organization and navigation
  For unfamiliar platforms, moderators need to add tutorial videos and organize content so that people who join in late or miss certain weeks can trace it back and respond. Potential workarounds include the following:
    Each week’s activity can be on a separate channel or group.
    Screen record tutorial videos and talk through functionalities such as threads, channels, and formatting

The internet-based asynchronous group format introduces *a moderation burden,* both in terms of time and effort related to monitoring for crisis support and adverse events. This was a concern for most clinicians and was also prevalent in volunteer-moderated internet-based groups [39]. To support participants in reaching moderators efficiently, the platform must have clear, discoverable methods for teens to connect with individuals moderating a group. Chatbots can also be designed to assist moderators by flagging content that needs to be reviewed by moderators and respond urgently.

### Conclusions and Future Work

Our study supported gaining in-depth feedback from a small number of participants for 10 weeks, including feedback on low-fidelity prototypes. Our findings should be interpreted in this context, including its limitations. Specifically, we recruited teens with mild-to-moderate depression and who had experience with therapy. This may have biased our results toward a positive attitude toward therapy compared with teens who may have issues with accessing therapy or who prefer self-support. In total, 2 clinicians and 4 teens eventually dropped out of the study by week 9; thus, we also do not know if those participants would have expressed different preferences in the activities they did not experience. Although participant perceptions about these mock-ups and their experiences indicate design needs and promising directions, future work should implement and evaluate these studies to assess whether and how they work in everyday practice and identify further opportunities to iterate on the design concepts presented here.

In our study, both teens and clinicians wanted to leverage the advantages of increased access to mental health care through an internet-based platform that supports an ARC approach to intervention delivery. The ARC model can act either as a supplement to face-to-face or telehealth-delivered therapy by allowing adolescents to stay engaged between visits or as a way of exclusively delivering therapeutic strategies, which can help increase the reach and access of EBPIs. We believe that the use of ARCs can be extended to other EBPIs beyond BA, although this remains to be evaluated in future work. Different care models may lend themselves particularly well to the integration of the ARC format for treatment delivery with traditional therapy, including stepped care [40], supplemented traditional care, and after-treatment care. For example, supplemented traditional care could include conducting weekly internet-based modules on a platform such as Slack during traditional therapy to decrease the number of in-person treatment sessions and support teens in their therapy goals, help with homework completion, and answer questions between in-person sessions.

Through our work, we highlight the need to integrate and support human infrastructure and digital technologies for teenage mental health. By following a human-centered co-design process with adolescents and clinicians, we have designed a delivery approach to directly engage with stakeholders and ensure that the design is well-grounded in the needs and constraints of those who will benefit from its use. By involving teens and clinicians early in the design process and presenting an empirical understanding of their needs, we hope to reduce the gap in navigating design tensions in internet-based and accessible mental health care. With the increase in mental health difficulties and the need to adapt research methods to remote format during COVID-19 quarantine, the ARC method has been useful in reaching a population of interest with minimal burden to both researchers and participants.

## Appendix

Multimedia Appendix 1 Internet-based group guidelines.

Multimedia Appendix 2 Adverse events protocol.

Multimedia Appendix 3 Protocol for reported child abuse.

Multimedia Appendix 4 Summary of behavioral activation sessions.

Multimedia Appendix 5 Study protocols.

Multimedia Appendix 6 Codebook.

## Abbreviations

ARC: asynchronous remote community
BA: behavioral activation
EBPI: evidence-based psychosocial intervention
HIPAA: Health Insurance Portability and Accountability Act
PHQ-8: Patient Health Questionnaire-8
SMART: specific, measurable, appealing, realistic, and timebound
