# Supplementary material for: Designing Asynchronous Remote Support for Behavioral Activation in Teenagers With Depression: Formative Study
Source: JMIR Form Res. 2021 Jul 13;5(7):e20969. doi: 10.2196/20969 (PMC8317030; doi:10.2196/20969)
Supplement: Multimedia Appendix 1 [file formative_v5i7e20969_app1.pdf]

Engaging Teenagers in Asynchronous Online Groups to Design for Stress Management

Appendix D: Online Group guidelines

--- These guidelines will be pinned to our Slack group ---

Hi! This is a private Slack group for a research study on designing tools for health management for adolescents. This group is mainly for research on understanding stressful factors in adolescent life and strategies for coping with stress. Everything you share on this group will be used for research purposes and is accessible to other members in the group. Please only share what you are comfortable with.

Please remember the following guidelines for the group:

- We honor **confidentiality.** Please do NOT share any information about members in the group outside the group.
- If you have questions or want to post something **anonymously**, you can message or email one of the moderators and we will post it without identifying you.
- Please respect other people's **privacy and personal space.** If someone does not want to add another person to their personal Facebook page as a "friend", please respect their decision.

Privacy guidelines:

- Please **do NOT share any personal health information or medical records** here (e.g.; pictures of prescriptions or medical reports) . Please be aware at all times that your information can be seen, downloaded, and/or saved as a screenshot by other participants in the group.

- Please **do NOT post any identifiable information** such as account number, full name, address, photographs of individuals in settings which are not public. You may blur the identifiable features before sharing or ask us to do so on your behalf. Please be aware that all images and videos are downloadable and/or people can take screenshots. Even though our research team will be careful to not share this information outside the study team, we do not have control over what other participants can share outside of this group.

- You may "block" individuals if you do not wish to see their posts and/or do not want them to see their posts. But do notify the research team if you decide to do so.

If you have any questions on privacy settings, feel uncomfortable, distressed, harassed, please contact the moderator(s).

Here is the privacy policy for Slack: https://slack.com/privacy-policy

Cite as: Arpita Bhattacharya, Calvin Liang, Emily Y Zeng, Kanishk Shukla, Miguel Rios Wong, Sean A. Munson, Julie A. Kientz. 2019. Engaging Teenagers in Asynchronous Remote Groups to Design for Stress Management. In *Proceedings of the 2019 Conference on Interaction Design and Children* (IDC '19). ACM, New York, NY, USA. DOI: https://doi.org/10.1145/3311927.3323140

To ensure that this group is a safe space for discussion on sensitive topics:

The purpose of discussions is not to come to any consensus, there are no wrong answers, and we may have disagreements. We encourage group members to listen to differing perspectives with an open mind.

- We honor diversity of opinions. **Respectful disagreement** is welcome. However, personal insults, biases and abusive language are not welcome. Use of such language may result in you being eliminated from the group and the study.
- Please be respectful of another individual's culture, beliefs, customs, and spirituality.
- If you sense that a person may be in crisis or posts something on the group that concerns you, notify the moderators immediately. We will follow up with necessary measures for support for you and the person of concern.

If you feel unsafe or in crisis, please contact for professional help:

- Crisis Text Line number: 741741 text START or HELP
- NATIONAL SUICIDE LIFELINE **1-800-273-TALK**
- Crisis clinic (24x7): 866-427-4747 / **206-461-3222 (King County)**
- Teen Link: **866-833-6546 (6-10 pm)**
- Link to other resources for support (See resource sheet)

We are all peers and want to be supportive of each other. We are NOT professionals. Peer advice and sharing from experiences is welcome, but please always check with your physician/psychiatrist/therapist, especially for medication and emergency situations.

Moderators are here to help:

- If there is a post that is disrespectful or violates above guidelines, please report it to the moderators, and they may delete it after discussion with the concerned individuals and research team.
- If someone feels they have been treated unfairly on the group or in private messaging with group members or receiving unwanted messages, you can bring it to our attention.
- Please **do not spam** the group page with offensive or inappropriate content or commercial advertising. Such content will be removed.

We encourage participation from all. However, failure to abide by our shared guidelines will result in a warning from the moderators. Your first warning will come from one of us, second warning will come after discussing with the research team. After the second warning, if further concerns arise, the person will be removed from the online group (while we do not hope that should happen).

Let the moderators know if you have questions or want to discontinue the study at any time.

Cite as: Arpita Bhattacharya, Calvin Liang, Emily Y Zeng, Kanishk Shukla, Miguel Rios Wong, Sean A. Munson, Julie A. Kientz. 2019. Engaging Teenagers in Asynchronous Remote Groups to Design for Stress Management. In *Proceedings of the 2019 Conference on Interaction Design and Children* (IDC '19). ACM, New York, NY, USA. DOI: https://doi.org/10.1145/3311927.3323140
