# Supplementary material for: Designing Asynchronous Remote Support for Behavioral Activation in Teenagers With Depression: Formative Study
Source: JMIR Form Res. 2021 Jul 13;5(7):e20969. doi: 10.2196/20969 (PMC8317030; doi:10.2196/20969)
Supplement: Multimedia Appendix 2 [file formative_v5i7e20969_app2.pdf]

Engaging Teenagers in Asynchronous Online Groups to Design for Stress Management

Appendix E: Adverse Events Protocol for Research Team

Emergency contact: All participants (including adolescents enrolled without parental permission) are required to provide emergency contact information for an adult in their network. The PI will reach out to this emergency contact under following circumstances:

(1) Disclosure of physical harm to self (including medical emergencies)
(2) Disclosure of physical harm to another individual.
(3) Disclosure of a minor in abuse.

All participants are and will be informed about exceptions to confidentiality in their respective consent and assent forms. Protocol for disclosures of emergency situations are explained below.

Involving parents: An emergency contact is an adult in the minor's network (e.g., parent, sibling, aunt, uncle, significant other). In the following scenarios, if the emergency contact is a parent, we will be able to reach them directly. If the emergency contact is not a parent, we will only work with the emergency contact s explained, encourage them to inform the parent, and we will not contact the parent directly.

Protocol: While we do not directly ask participants about situations of minor in abuse or intent for suicide, this protocol ensures that the research personnels will be prepared for any unexpected events at all times.

Management of Emergency Situations
During the conduct of research with human participants, there is always the potential that an emergency situation will arise. The general categories of emergency situations that might arise include:
(1) medical emergencies,
(2) disclosure of suicidal ideations
(3) disclosure of minor in abuse
(4) emotional distress: grievances of online harassment, bullying, hate speech, violating group guidelines,
(5) privacy or confidentiality breach in online group setting

Constraints and workarounds due to online and asynchronous nature of the study:

1. In this study, all procedures are conducted over phone, email, or online Facebook/Slack groups. We will not be in physical proximity of participants or aware of their physical location to be able to assist them on the spot. Therefore, in addition to contact information of the participants, we are obtaining contact information of an adult emergency contact person for each participant on their consent forms, so we can reach out to them in case of emergency.
2. **For issues posted on the online group, private messages, emails,** we are not staffed to be able to monitor situations 24x7. We have provided contact information for appropriate resources for adverse events to participants in all consent/assent forms and in Appendix F (Group Guidelines). Appendix F will be available as a pinned post on every Facebook/Slack online group.
3. We will monitor online group activities at least once every business day and will be able to reach out to the participant as soon as we learn of the emergency within that business day.
4. PI will provide hours when participants can call in with issues directly on business days, depending on her schedule for the quarter. This will be also posted on the pinned guidelines.
5. Study personnels will attempt to involve the participant in decision making as much as possible.

Our maximum turn-around-time to make first contact with the participant regarding an issue will be within 24 hours on business days.

Cite as: Arpita Bhattacharya, Calvin Liang, Emily Y. Zeng, Kanishk Shukla, Miguel Rios Wong, Sean A. Munson, Julie A. Kientz. 2019. Engaging Teenagers in Asynchronous Remote Groups to Design for Stress Management. In *Proceedings of the 2019 Conference on Interaction Design and Children* (IDC '19). ACM, New York, NY, USA. DOI: https://doi.org/10.1145/3311927.3323140

The following guidelines are designed to support project personnel in the management of emergency situations. **We are not providing any counselling and/or medical service.** Specifically, these policies and procedures are designed to assist project personnel with identifying and directing participants to resources that are the most appropriate for providing crisis or emergency services in specific instances.

As soon as study personnel is aware of emergency situations, they should contact Principal Investigator (PI) (**arpitab@uw.edu**/ 323-686-1624) and faculty advisors as soon as possible, so that they can assist in the management of the emergency situation.

In cases where we break confidentiality resulting from these emergencies, PI will inform the HSD.

(1) MEDICAL EMERGENCIES

As the study is online, we do not expect physical injuries or emergencies due to the study. In case the participant describes or discloses a current event leading to medical emergency to the study personnel or on the online group (e.g. cardiac arrest, sudden loss of consciousness):

1. First, try to **reach the participant**. If already in contact with the participant or able to make contact with the participant, the project personnel should ask the participant to immediately dial **911** on the nearest phone.

2. The study personnel should also **call the participant's emergency contact** provided on the consent form as soon as possible and inform the contact person about the disclosure and context of emergency. Ask them to dial 911 if the participant is not able to do so.

3. **If neither the participant nor their emergency contact is reachable, study personnel should dial 911** themselves and inform the police about the emergency and contact information of the participant and their emergency contact with details that is available with the research team.

4. PI will inform the IRB about the emergency and the reason for breaking confidentiality.

---

(2) DISCLOSURE OF SUICIDAL IDEATIONS

For this study, research personnels will not explicitly ask participants if they are experiencing suicidal ideations. However, if participants express current thoughts of suicide or death or in response to any study tasks on the online group or private correspondence: as soon as the study team is made aware of this, contact the PI (**arpitab@uw.edu**/ 323-686-1624) so she can contact the participant.

Protocol for PI/trained staff:

1. First, try to **reach the participant**. If already in contact with the participant or able to make contact with the participant, inquire about the situation by asking, "Are you thinking about suicide?" Listen to the participant's response.

2. If participant says yes to the above, the study personnel will **first ask the participant if they have a mental health therapist or counselor they can reach out to within the next 24 hours.** If yes, encourage them to reach out to that professional for support within the next 24 hours.

3. Regardless of whether they have a therapist or not, **also provide the participant with the following resources (verbally and either email or text)** during the conversation, by emphasizing

Cite as: Arpita Bhattacharya, Calvin Liang, Emily Y. Zeng, Kanishk Shukla, Miguel Rios Wong, Sean A. Munson, Julie A. Kientz. 2019. Engaging Teenagers in Asynchronous Remote Groups to Design for Stress Management. In *Proceedings of the 2019 Conference on Interaction Design and Children* (IDC '19). ACM, New York, NY, USA. DOI: https://doi.org/10.1145/3311927.3323140

that, "these are resources where professionals can help", and encourage them to reach out to these resources for support:

National Suicide Prevention Lifeline (24x7): 1-800-273-8255
Crisis Line (24x7): 1- 866-427-4747
Teen peer support: TeenLink: https://866teenlink.org/ 1-866-TEENLINK
Crisis text line: 741741 -- text HOME/HELP

4. Next, ask the participant **if there is an adult in their social circle who they want to reach out who can help them feel safe** (if it is a minor, emphasize that they pick an adult contact person). If so, ask the participant if they want help reaching out to that person and provide that help if requested (e.g.; dialing the number and staying on call/chat with them). As indicated and with permission from the participant, study personnel should facilitate the contact between the subject and the emergency contact or between the subject and the Crisis Line.

5. **If the participant declines that help, nonetheless, contact the participant's emergency contact** (provided on the consent/assent form) to let the contact person know that the participant is experiencing thoughts of suicide and explain to them the context of disclosure. All participants are explained in the consent form that we will break confidentiality in situations of threat to self/others.

6. Provide the emergency contact person with the helpline numbers in step 3, and let them know that they can themselves call the helpline numbers where crisis helpline professionals can help them prepare a safety plan over the phone.

7. **In cases where PI is unable to reach the participant after disclosure**, she will directly contact the subject's emergency contact (provided on the consent/assent form) to let the contact person know about the disclosure and context of disclosure and repeat step 5 and 6.

8. **If neither the participant nor their emergency contact is reachable, and the last post or message from the participant is directly indicative of imminent threat to self/others: study personnel should contact the clinical staff,** and consult with them if it is required to dial 911 to inform the police about the emergency and contact information of the participant and their emergency contact with details that is available with the research team.

9. PI will inform the IRB about the emergency and the reason for breaking confidentiality.

Facebook has also provided links for Suicidality and Self-Injury resources, which the study personnel can provide to the participant or use as reference:
https://www.facebook.com/help/1553737468262661/?helpref=hc_fnav

---

(3) DISCLOSURE OF MINOR IN ABUSE

We are not directly asking participants if they or any minor is in abuse. However, if participants disclose a situation where a minor is in abuse, following actions need to be taken:

1. Inform the PI, who is a mandatory reporter. PI will work with clinical psychologists on the team to determine next steps. The protocol for reported child abuse is explained in Appendix I as developed by our team members who are clinical psychologists.

Cite as: Arpita Bhattacharya, Calvin Liang, Emily Y. Zeng, Kanishk Shukla, Miguel Rios Wong, Sean A. Munson, Julie A. Kientz. 2019. Engaging Teenagers in Asynchronous Remote Groups to Design for Stress Management. In *Proceedings of the 2019 Conference on Interaction Design and Children* (IDC '19). ACM, New York, NY, USA. DOI: https://doi.org/10.1145/3311927.3323140

2. PI will contact the person who disclosed the situation of abuse and further inquire about it. If it is a minor in abuse, PI will aim to obtain the following contact information to report to Child Protection Services of their respective state within the time window of that state (e.g.; within 48 hours for WA).
   Child:
   a. Name
   b. Address
   c. Phone
   Parent:
   d. Name
   e. Address
   f. Phone
   Perpetrator:
   g. Name
   h. Contact information
   i. Description of event
3. Depending on the situations (described in Appendix I) and whether the perpetrator is same as the parent contact or not, the PI will attempt to contact and work with an adult family member/emergency contact of the minor to inform them and assist with creating a safety plan after consulting our clinical staff. Child's safety will be of utmost priority.
4. PI will inform the IRB if and when we break confidentiality.
5. **If it is an adult in abuse**, PI will provide list of resources that the adult can reach out to for assistance and encourage them to contact the resources. In cases of an adult in abuse, it is ultimately the participants decision to take action or not and we will not break any confidentiality.

(4) EMOTIONAL DISTRESS: GRIEVANCES OF ONLINE HARASSMENT, BULLYING, HATE SPEECH, VIOLATING GROUP GUIDELINES

Study personnel will have admin rights to online groups. Admins can delete posts that violate Group Guidelines (Appendix F). These posts include text/images of harassment, bullying, disrespectful language towards any community, religious beliefs or group, perpetuating/using hate speech and/or physical violence towards another human being, spam or commercial advertisements.

When a study personnel identifies such a post or a participant reports a grievance based on the above:

1. Take a screenshot of the post or comment thread and save it in password protected device.
2. Contact the PI and other members of the study team to discuss if the post needs to be deleted.
3. Delete the post/comment or take actions based on the team's decision.
4. Contact the participants involved in the post/comment through private message. Inquire about the situation to clarify if we need more information and explain the reason to the involved participants for deleting or not deleting the post/comment.

When the study team is made aware of a situation of emotional distress:

5. We will respond to the participants involved within 24 hours on business days, and clarify what the situation is: Inquire if the participant(s) need any emotional support and if so, encourage them connect with an adult in their network for support and/or professional (such as school's counseling

Cite as: Arpita Bhattacharya, Calvin Liang, Emily Y. Zeng, Kanishk Shukla, Miguel Rios Wong, Sean A. Munson, Julie A. Kientz. 2019. Engaging Teenagers in Asynchronous Remote Groups to Design for Stress Management. In *Proceedings of the 2019 Conference on Interaction Design and Children* (IDC '19). ACM, New York, NY, USA. DOI: https://doi.org/10.1145/3311927.3323140

service). If this is not an immediate threat to self or other (not involving suicidal ideations or physical violence), study personnel should honor confidentiality and not contact anyone other than the involved participant regarding this matter.

6. If it is an emergency, we will inform the participants to contact 911 or a crisis hotline
7. If it is not an emergency, the study team will direct the participant towards the appropriate resources. The study team will not provide counseling but will inform the participant about the availability of resources
8. PI will inform the IRB if any identifiable information of any participant is compromised due to this.

Resources from Facebook on bullying and abuse: Participants will not be asked to report abuse through Facebook as it will compromise confidentiality. However, study team will have these resources provided by Facebook for reference in dealing with these online grievances.
This is for teens: https://www.facebook.com/safety/bullying/teens
Reporting abuse: https://www.facebook.com/help/1417189725200547/
How to report things: https://www.facebook.com/help/reportlinks

---

(5) PRIVACY AND CONFIDENTIALITY BREACH IN ONLINE GROUP SETTING

We are not seeking to obtain any identifiable information about third-party subjects **on the online group where other participants in the group can also view or access the information.** In the event that a subject posts identifiable data (image or text) about themselves or a third party on the online group, study personnel should either:

(A) request that the subject remove those data by explaining them the reason for protecting privacy; or

(B) remove the data themselves and inform the participant on private chat or email what data was removed and reason for removal.

All participants in a group will be requested to honor confidentiality and privacy of other participants as explained in the pinned Group Guidelines (Appendix F). Through Appendix F, participants will also be informed to not post any identifiable information on the group. In case it is alleged or proven that a participant has deliberately violated privacy/confidentiality of another participants' information in the group setting,

1. the study personnel should contact all participants involved in the alleged confidentiality/privacy breach and inquire about the situation,
2. Discuss the case among research team with faculty advisors for planning next steps.
3. PI will inform the IRB if any identifiable information of any participant is compromised due to this.

Cite as: Arpita Bhattacharya, Calvin Liang, Emily Y. Zeng, Kanishk Shukla, Miguel Rios Wong, Sean A. Munson, Julie A. Kientz. 2019. Engaging Teenagers in Asynchronous Remote Groups to Design for Stress Management. In *Proceedings of the 2019 Conference on Interaction Design and Children* (IDC '19). ACM, New York, NY, USA. DOI: https://doi.org/10.1145/3311927.3323140
