# Supplementary material for: Designing Asynchronous Remote Support for Behavioral Activation in Teenagers With Depression: Formative Study
Source: JMIR Form Res. 2021 Jul 13;5(7):e20969. doi: 10.2196/20969 (PMC8317030; doi:10.2196/20969)
Supplement: Multimedia Appendix 3 [file formative_v5i7e20969_app3.pdf]

Engaging Teenagers in Asynchronous Online Groups to Design for Stress Management

Appendix F: Protocol for Disclosures of Child Abuse

Emergency contact: All participants (including adolescents enrolled without parental permission) are required to provide emergency contact information for an adult in their network. The PI will reach out to this emergency contact under following circumstances:

(1) Disclosure of physical harm to self (including medical emergencies)
(2) Disclosure of physical harm to another individual.
(3) Disclosure of a minor in abuse.

All participants are and will be informed about exceptions to confidentiality in their respective consent and assent forms. Protocol for disclosures of minor in abuse is explained below.

Involving parents: An emergency contact is an adult in the minor's network (e.g., parent sibling, aunt, uncle, significant other). In the following scenarios, if the emergency contact is a parent, we will be able to reach them directly. If the emergency contact is not the parent and we or the Child Protection Services (CPS) need to reach the parent(s), as explained below, we will work with the child and/or the emergency contact to obtain contact information of the parent. We will only contact the parent if they are not an emergency contact as directed by our clinical staff and CPS and inform the HSD about it.

Protocol:
In the state of Washington, there is a legal obligation to report current and/or past sexual or physical abuse of a minor, regardless of how long ago the abuse occurred. We are all mandated reporters.
http://app.leg.wa.gov/rcw/default.aspx?cite=26.44.030

**In the case of any reported abuse, contact the clinical back-up (Dr. Jenness and/or Dr. McLaughlin) as soon as study personnel/PI is made aware of the disclosure of situation of abuse. . In cases other than situations where CPS and parent are both aware of the abuse, contact clinical back up to assist with disclosure, reporting and safety planning as soon as possible.

- Criteria for physical or sexual abuse:
    - For a definition of neglect, physical abuse, and sexual abuse, see p.3-4 of "Protecting the Abused and Neglected Child" in the References section below. Basic definitions attached as final page of this document
- Priorities when presented with a case of:
    - Child safety
    - Gathering information about the event and parties involved
    - Law and Reporting

Cite as: Arpita Bhattacharya, Calvin Liang, Emily Y Zeng, Kanishk Shukla, Miguel Rios Wong, Sean A. Munson, Julie A. Kientz. 2019. Engaging Teenagers in Asynchronous Remote Groups to Design for Stress Management. In *Proceedings of the 2019 Conference on Interaction Design and Children* (IDC '19). ACM, New York, NY, USA. DOI: https://doi.org/10.1145/3311927.3323140

Research team members who do not have clinical training, should contact clinical back-up as soon as they can and inform them of the disclosure and context of the disclosure, and relevant contact information of the participants, legal guardian, and emergency contact available with research personnel. PI will discuss the case with clinical back-up and inform CPS within the timeframe of reporting as required by the minor's state.

Clinical professionals on our study team have developed the following protocol and study personnel/PI should assist them in collecting that information from participants, if and when required. **(Note: This protocol provides instructions so study personnel is aware what actions may be taken, please do not take steps on your own without consulting with clinical staff)**:

Current Abuse

Scenario 1: Child is being abused by parent or caregiver (in household) and no CPS report has been made.

1. If the child reports present abuse, ask follow up questions:
    i. Does the child feel in danger?
    ii. Does the child feel scared?
    iii. What type of abuse—physical and/or sexual What happened?
    iv. Has the abuse happened more than once? If so, how often?
    v. How many times was the child hit and with what object (fist, belt, open hand, etc.)?
        a. Were there any bruises or marks left from being hit?
    vi. Was the parent under the influence of drugs or alcohol?
    vii. Try to obtain as much information as possible including address and names of persons involved.
2. If the parent hit the child:
    i. Some parents may endorse spanking/corporal punishment when their child misbehaves. This may not be a good parenting strategy, but such punishment does not necessarily rise to the level of abuse. To determine if it is a potential abuse situation, ask the child how often it happens, under what circumstances, and if there were any bruises or marks.
    ii. Discuss with clinical back-up whether the case is corporal punishment or abuse – if abuse a report must be made.
3. Make a plan for Child Safety:
    i. What will happen to the child when the parent finds out that the child disclosed the abuse?
    ii. Determine if it is safe for the child to go home.
        a. If not, identify potential places where child can stay (i.e., family members, friends) assuming the parent who is the contact person on the

Cite as: Arpita Bhattacharya, Calvin Liang, Emily Y Zeng, Kanishk Shukla, Miguel Rios Wong, Sean A. Munson, Julie A. Kientz. 2019. Engaging Teenagers in Asynchronous Remote Groups to Design for Stress Management. In *Proceedings of the 2019 Conference on Interaction Design and Children* (IDC '19). ACM, New York, NY, USA. DOI: https://doi.org/10.1145/3311927.3323140

permission form/ emergency contact or caregiver in household is the abuser.

b. Try to involve parent (if parent who is the contact person on the permission form/emergency contact is not the abuser) as much as possible in safety planning.

c. CPS may need to transport child to alternative care.

iii. Parent may be hostile/upset, may need to separate parties to deescalate the situation; if you feel in danger ask for backup from staff and contact the police if necessary

4. Inform family and youth that CPS report will be made immediately:
   i. The best outcome would be if the parent calls CPS with the researcher or our assistance.
      a. If in this situation, try to present yourself as being on the side of the family—you are required by law to do this and you recognize that it will be really hard for the family.
      b. If parent leaves abruptly with child contact CPS immediately.

Scenario 2: Current Abuse **not** by Parent/Caregiver – neither parent nor CPS knows about abuse.

1. Ask follow-up questions in #1.
2. Must report to CPS.
3. Create safety plan with parent:
   i. Does child need to stay at a different house?
   ii. Does the child need to change schools or their commute to/from home?
   iii. Domestic violence shelter? Neighbors? Friends?
   iv. Parent **should** be involved in making the report to CPS.

Note: The parent may become distressed and upset in learning about past or present abuse (angry that the event occurred, that they never knew, etc.). Reduce parental distress and normalize the child's reaction to their upset parent/the abuse.

Scenario 3: Current abuse not by parent – Parent is aware of abuse, but no CPS report.

1. This case is extremely rare, but must file CPS report.
2. Gather information regarding who, what, when, where, and how the child was abused. Information about the abuser is helpful and should be obtained if possible:
   i. Name
   ii. Phone number
   iii. Address

Cite as: Arpita Bhattacharya, Calvin Liang, Emily Y Zeng, Kanishk Shukla, Miguel Rios Wong, Sean A. Munson, Julie A. Kientz. 2019. Engaging Teenagers in Asynchronous Remote Groups to Design for Stress Management. In *Proceedings of the 2019 Conference on Interaction Design and Children* (IDC '19). ACM, New York, NY, USA. DOI: https://doi.org/10.1145/3311927.3323140

3. If it does occur, follow the procedures above for debriefing the child and parent about why there must be a report filed to CPS.

<u>Scenario 4</u>: Parent and CPS know about past or current abuse.

1. Verify with parent this is accurate
2. If parent confirms, you do not need to report.
3. Document on session checklist that report has been made with CPS and parent confirms.

Previous Abuse

<u>Scenario 5</u>: Neither the parent nor CPS knows about the past abuse.

1. Talk with the child about why you have to break confidentiality:
    i. Discuss how the child wants to tell the parent – find out if he/she is comfortable or would rather you tell the parent. If child prefers to tell parent it must be in presence of research staff.
    ii. Prepare the parent for what their child is about to tell them (or what you are about to tell the parent). Start with the least amount of detail.

Note: The parent may become distressed and upset in learning about past or present abuse (angry that the event occurred, that they never knew, etc.). Reduce parental distress and normalize the child's reaction to their upset parent/the abuse.

2. Discuss with parent how the reporting should happen; e.g. whether they want to report it with you or if they would prefer you to report it.

3. Make sure the child is safe and does not come in contact with abuser.

<u>Scenario 6</u>: Parent is aware of past abuse by other caregiver or adult, but Child Protective Services (CPS) does not know about the past abuse.

1. Explain to parent we are obligated to tell CPS; ask parent if they would like us to report it or if we should report the incident together.
2. Get as much information about perpetrator as possible and about what happened (see Reporting section about the specific information needed).
3. Report to CPS.
4. Create safety plan if child is in contact with abuser in any way.

Cite as: Arpita Bhattacharya, Calvin Liang, Emily Y Zeng, Kanishk Shukla, Miguel Rios Wong, Sean A. Munson, Julie A. Kientz. 2019. Engaging Teenagers in Asynchronous Remote Groups to Design for Stress Management. In *Proceedings of the 2019 Conference on Interaction Design and Children* (IDC '19). ACM, New York, NY, USA. DOI: https://doi.org/10.1145/3311927.3323140

Scenario 7: Parent who is the contact person on the emergency contact previously abused child and CPS does not know.

1. Follow steps as listed in scenario 1 that parent is currently abusing child.

Reporting to CPS (to be done by clinical back up)

Daytime - Find your local office number to report abuse or neglect in your area.

Nights & Weekends - call **1-800-562-5624** to report abuse during the evening or on weekends.

Hotline - call **1-866-ENDHARM**

Any reports to CPS should be made within 48 hours of the child's visit to the lab.

- Child:
  - Name
  - Address
  - Phone
- Parent:
  - Name
  - Address
  - Phone
- Perpetrator:
  - Name
  - Contact information
  - Description of event

IRB:

- Any cases of abuse also have to be reported to the IRB by the PI.

RCW 26.44.020

Definitions.

1) "Abuse or neglect" means sexual abuse, sexual exploitation, or injury of a child by any person under circumstances which cause harm to the child's health, welfare, or safety, excluding conduct permitted under RCW 9A.16.100; or the negligent treatment or maltreatment of a child by a person responsible for or providing care to the child. An abused child is a child who has been subjected to child abuse or neglect as defined in this section.

(16) "Negligent treatment or maltreatment" means an act or a failure to act, or the cumulative effects of a pattern of conduct, behavior, or inaction, that evidences a serious disregard of

Cite as: Arpita Bhattacharya, Calvin Liang, Emily Y Zeng, Kanishk Shukla, Miguel Rios Wong, Sean A. Munson, Julie A. Kientz. 2019. Engaging Teenagers in Asynchronous Remote Groups to Design for Stress Management. In *Proceedings of the 2019 Conference on Interaction Design and Children* (IDC '19). ACM, New York, NY, USA. DOI: https://doi.org/10.1145/3311927.3323140

consequences of such magnitude as to constitute a clear and present danger to a child's health, welfare, or safety, including but not limited to conduct prohibited under RCW 9A.42.100. When considering whether a clear and present danger exists, evidence of a parent's substance abuse as a contributing factor to negligent treatment or maltreatment shall be given great weight. The fact that siblings share a bedroom is not, in and of itself, negligent treatment or maltreatment. Poverty, homelessness, or exposure to domestic violence as defined in RCW 26.50.010 that is perpetrated against someone other than the child does not constitute negligent treatment or maltreatment in and of itself.

RCW 9A.16.100

Use of force on children — Policy — Actions presumed unreasonable.

It is the policy of this state to protect children from assault and abuse and to encourage parents, teachers, and their authorized agents to use methods of correction and restraint of children that are not dangerous to the children. However, the physical discipline of a child is not unlawful when it is reasonable and moderate and is inflicted by a parent, teacher, or guardian for purposes of restraining or correcting the child. Any use of force on a child by any other person is unlawful unless it is reasonable and moderate and is authorized in advance by the child's parent or guardian for purposes of restraining or correcting the child.

The following actions are presumed unreasonable when used to correct or restrain a child: (1) Throwing, kicking, burning, or cutting a child; (2) striking a child with a closed fist; (3) shaking a child under age three; (4) interfering with a child's breathing; (5) threatening a child with a deadly weapon; or (6) doing any other act that is likely to cause and which does cause bodily harm greater than transient pain or minor temporary marks. The age, size, and condition of the child and the location of the injury shall be considered when determining whether the bodily harm is reasonable or moderate. This list is illustrative of unreasonable actions and is not intended to be exclusive.

References:
Collect Information:

- http://app.leg.wa.gov/rcw/default.aspx?cite=26.44.040

- Health and Human Services PPT:
  - http://www.acf.hhs.gov/sites/default/files/fysb/mandatory_reporting_508.pdf
- Protecting the Abused and Neglected Child:
  - http://www.dshs.wa.gov/pdf/publications/22-163.pdf
  - http://app.leg.wa.gov/rcw/default.aspx?cite=26.44.030

Clinical Backup: Who to Contact

When contacting clinical staff start with Dr. Jenness and move down the list: call first; if no answer, then text; if still no answer, then email.

Cite as: Arpita Bhattacharya, Calvin Liang, Emily Y Zeng, Kanishk Shukla, Miguel Rios Wong, Sean A. Munson, Julie A. Kientz. 2019. Engaging Teenagers in Asynchronous Remote Groups to Design for Stress Management. In *Proceedings of the 2019 Conference on Interaction Design and Children* (IDC '19). ACM, New York, NY, USA. DOI: https://doi.org/10.1145/3311927.3323140

1st Contact:

- Dr. Jessica Jenness
- [Phone number]
- jennessj@uw.edu

Cite as: Arpita Bhattacharya, Calvin Liang, Emily Y Zeng, Kanishk Shukla, Miguel Rios Wong, Sean A. Munson, Julie A. Kientz. 2019. Engaging Teenagers in Asynchronous Remote Groups to Design for Stress Management. In *Proceedings of the 2019 Conference on Interaction Design and Children* (IDC '19). ACM, New York, NY, USA. DOI: https://doi.org/10.1145/3311927.3323140
