# Supplementary material for: Designing Asynchronous Remote Support for Behavioral Activation in Teenagers With Depression: Formative Study
Source: JMIR Form Res. 2021 Jul 13;5(7):e20969. doi: 10.2196/20969 (PMC8317030; doi:10.2196/20969)
Supplement: Multimedia Appendix 4 [file formative_v5i7e20969_app4.pdf]

APPENDIX : Summary of BA sessions - short (4-6 weeks)

Session	Description	Example
**Week 1: Introduction to Behavioral Activation (BA)**	● Introduce the BA model of depression and treatment.<br><br>● Review how situations, feelings, and actions lead to downward or upward spiraling of mood.<br><br>● Introduce tracking activities and how you feel (mood) during or after each activity throughout the day.	1. BA Model example: Stressful situations can lead to negative mood and behaviors that make things harder to feel better (e.g., staying in your room more, not doing homework).<br><br>2. Those negative behaviors may lead to more problems/stress which results in *a downward spiral* towards low mood/depression.<br><br>Upward spirals: When your actions follow a *goal* instead of your *mood* to help improve how you feel

**Week 2: Goal Setting**	• Talk through how to set SMART goals (Specific, Measurable, Appealing, Realistic, and Time-Bound) related to improving mood<br><br>• Introduce importance of breaking down the goals into smaller steps, i.e., mini-steps to reach a goal.<br><br>• Practice setting up SMART goals and mini-steps for the week for yourself.	1. SMART Goal Example: I plan to go on a 30-min walk with my dog and friend 3x this week<br><br>2. Mini-steps: 1) Check the weather to find the best days; 2) text my friend to see which days work; 3) pick a park or walking route; 4) send myself/my friend a google calendar invite

**Week 3: Identifying Barriers & Problem Solving**	● Importance of identifying internal and external barriers that get in the way of accomplishing goals.<br><br>● Introduce problem-solving to help overcome barriers<br><br>● Practice goal-setting.	1. Examples of Barriers:<br><br>*Internal barriers* include not feeling motivated, getting distracted, difficulty communicating your needs, feeling overwhelmed.<br>*External barriers* include not having the right "tools" (e.g., need exercise equipment from a gym), need help from others (e.g., need a ride), other people not following through, or cost.<br><br>2. Problem-solving example: When encountering a barrier, you can use *COPE*: Calm yourself then Clarify the issue, brainstorm Options, Pick and Perform an option, Evaluate how it went.

**Week 4. Overcoming Avoidance**	• Explaining that wanting to avoid something (avoidance) is a common internal barrier.<br><br>• Understanding your Trigger, Response, and *Avoidance Pattern* (TRAP).<br><br>• Using *Alternative Coping* to get back on TRAC(k).<br><br>.	1. Examples of Avoidance: *Brooding* over a problem instead of solving it, *Procrastinating* taking action, *Hibernating* in your room or withdrawing socially, *Bursting* at people out of anger.<br><br>2. Example of TRAP/TRAC(k): I feel triggered when my mom yells at me to clean my room, I respond by feeling angry and worthless, and I have an *Avoidance Pattern* of yelling back and starting a fight. I will try<br><br>*Alternative Coping* of putting on my favorite dance song and listening to a podcast while I pick up my room.
**Week 5 & 6: Practice**	Practice setting SMART Goals and Overcoming Barriers to goals.	
