# Supplementary material for: Designing Asynchronous Remote Support for Behavioral Activation in Teenagers With Depression: Formative Study
Source: JMIR Form Res. 2021 Jul 13;5(7):e20969. doi: 10.2196/20969 (PMC8317030; doi:10.2196/20969)
Supplement: Multimedia Appendix 5 [file formative_v5i7e20969_app5.pdf]

Appendix: Study Protocols

- Weekly online activities: pages 1-10
- Interview protocol: pages 11-18

Weekly Online Activities

Week 1

Activities for Week 1:

--------------xxxxxxxxxxxx---

[**ACTIVITY: INTRODUCTIONS**]: Hi everyone @channel ! Thank you for your patience over the past week or so, we will now get started with the introductions! Keep in mind that each weekly activity will look similar to this one 😊

[Introductions]: Please introduce yourself to other members in the group. You can share using images or giphys as well.

1. Your **Slack name**
2. What is **one thing that helps bring your mood up**? This can be a hobby or something you like doing or watching!
3. What is **one coping strategy** that you have tried using when things are hard? Has this coping strategy been helpful?
4. **[Demographic Survey]:** Please fill out this demographic survey (~5 min): https://www.surveygizmo.com/s3/4896680/Teen-Demographic-survey-Teen-Depression-ARC

Week 2

[**ACTIVITY: TECHNOLOGY TO MANAGE MOOD**]: Hi @channel! This week's activity is on sharing and discussing tools and resources that you currently use to manage mood and depression. Please post a single message on this channel with your responses to the following questions:

(1) Examples of **digital technologies** that you have ever used in **therapy** or on **your own** (if any). You may post links, photos, or videos of what you use.

-- These tools may include but are not limited to apps (e.g., mood tracking apps, mindfulness apps, calendar apps), Fitbit or other tracking devices, websites, online videos, games, virtual reality, social media, and text messaging

(2) Examples of **non-digital tools or resources** you have ever used in **therapy** or on **your own**(if any). You may post links, photos, or videos of what you use.

-- These tools may include paper worksheets, book recommendations, in-person social or support groups, paper handouts or informational pamphlets

(3) If applicable, what are some ways you have **benefited** from using technology to manage mood and depression?

(4) If applicable, what are some ways that technology has been **unhelpful** or **challenging**?

(5) If you have not used any technological tools to manage mood and depression, or have stopped using any tools, please explain why

Please share what you are comfortable with. This activity should take about 20 minutes to complete. Hope you can complete it by **4/12 (Friday).**

Week 3

Poll:

Question: What has been the easiest way for you to ask for help when you are struggling with symptoms of depression or sadness?

- ***Face-to-face help from a friend or trusted person***
- ***Phone call to anonymous hotline***
- ***Phone call to a friend or trusted person***
- ***Texting friends or trusted person***
- ***Texting with strangers such as a helpline anonymously (e.g., Teenlink)***
- ***One-on-one online chat (anonymous)***
- ***Posting anonymously for peer support on social media groups (e.g., Reddit groups)***
- ***Contacting a mental health professional or trained volunteer via phone***

- ***Contacting a mental health professional or trained volunteer via text/email***
- ***Other***

Week 4

[ACTIVITY: Online Mental Health Support Part 2]: Hi @channel! Based on your feedback from last week on how a platform like Slack might be used for the delivery of depression treatment, we have put together ideas. There are *three parts* to this activity (A, B, and C).

1. *** Reactions to other ideas (~10 min)* -:** One strength of this platform is that you might be able to interact with other teens who are dealing with similar experiences.

- When posting about struggles with mood/depression, what kind of feedback or support would you like best from others on an online platform (e.g., advice, understanding/validation, encouragement)?
- Provide feedback and/or ask a follow-up question on another group members' post as a thread from last week or previous weeks. Please feel free to discuss and build on each others' ideas. As usual, you can express your thoughts in text, drawings, and/or images.

B. *Reflecting on receiving feedback (~10 min)*: Either based on experiences you've had so far or what you imagine ...

- What might be the benefits of being a part of an online group with other teens when receiving help for mood/depression?
- What might be the challenges or problems of being a part of an online group with other teens when receiving help for mood/depression?

C. Polls: We synthesized your ideas from the previous activity and have created a poll to understand what will be helpful features for online platforms to support mental health. Please vote on the the following polls. Please provide any additional explanation for your responses and/or add other options or comments in threads for each poll.

1: <u>Question: Thinking about individual vs group formats of therapy sessions, how do you think technology platforms such as Slack can play a role (you can select multiple options)?</u>

- **Online one on one support through technology between weekly in-person one on one therapy sessions with clinicians**

- **Support through one on one therapy but mostly online (alternative to in-person therapy)**
- **Support through online technology, between weekly in-person group sessions**
- **Support through online group therapy but mostly remotely (alternative to in-person therapy) moderated by a professional**

2: Question: <u>Which of the following options do you consider useful when thinking about how to best utilize an online platform such as Slack for treatment. Please select all that apply (setting aside privacy concerns and technological limitations of Slack)?</u>

- **Tracking mood, questionnaire responses, and viewing mood over time**
- **Tracking and personalized coaching support with sleep and other lifestyle changes (e.g., physical or social activity.)**
- **Using photos or videos for journaling or tracking mood or lifestyle for discussion at therapy sessions**
- **Getting automated reminders for therapy homework and appointments**
- **Homework delivery on the platform such as a document plugin**
- **Always-available bot for in-the-moment coping strategies (including redirecting to crisis helplines and problem solving)**
- **Always-available bot for venting and validation (i.e., place to express feelings/thoughts without judgment)**
- **Space for open-ended peer support and conversation (in group therapy format)**
- **Digital repository of Therapist-recommended apps and resources that can be updated**
- **Posting psychoeducational and training modules using videos**

<u>Week 5</u>

[ACTIVITY: Behavioral Activation]: Hi @channel! Thank you for all the valuable feedback so far on how technology can be used for depression treatment! Our project goal is to adapt a specific treatment, Behavioral Activation (BA), to an online platform. Research has shown that BA is an effective strategy for treating depression in both adults and teens.

BA comes from the idea that what you **_*do*_** affects how you **_*feel*_**. Because of this, BA focuses mostly how we can help people to **_*find*_** and **_*engage with*_** the activities and people in their lives that help improve mood!

There are 3 parts to this week's activity.

Part 1 (<7 min): Watch a video on BA You all have already given us lots of examples of how you use activities and people in your life to help you cope with feeling down. We'd like to make sure that we are all on the same page about what BA involves. *Please watch this ~7 minute video on the idea of Behavioral Activation (explanation starts at **0:47**):*

https://www.youtube.com/watch?v=1R6-gLZZhYc&feature=youtu.be&t=47

(**Note, this video is only meant as an introduction to how BA works, we are not using the worksheets or website mentioned in this video)

Part 2 (5 mins): Summary of the concepts of BA In the attached PDF, is a table of the concepts and skills taught during specific sessions of BA. For each concept and skill, we have also provided examples of what we mean. You will be learning more and trying out activities from each session in the coming weeks. We are aiming to create a 4-6 session treatment. Please review the table below: (Appendix A)

***Part 3: Post discussion comment (5-10 mins)*:** After watching the video and reviewing the summary table, please discuss the following by posting a comment on this thread:

(1) What are your reactions to using BA strategies to support improvement in mood and depression symptoms for teens? What aspects of these strategies make sense to you for help with mood and depression? (You can also write about what was confusing or unclear to you)

(2) What BA strategies or similar strategies have you tried either on your own or with the help of a therapist?

We hope you can complete this by Monday, May 6th.

Week 6

[ACTIVITY: Adapting BA]*: Hi @channel! Thank you for all of your feedback so far. We now plan on getting your feedback on some ways we can adapt Behavioral Activation on an online platform such as Slack!There will be two parts to this activity, *please provide feedback on the following technology adaptations*:*Part 1*: Introducing the BA model and how it works for you*1a.

Worksheet:* The first step is to explain the BA model and how it works. We typically do this in-person by talking through the model and asking you to fill out this worksheet: (edited)

1b. Technology adaptation: You can then fill this out yourself and upload a photo which can be reviewed by a clinician like this one.

Part 2: *Activity and Mood* tracking to better understand how your actions might impact mood in your daily life. *Please provide feedback on the following technology adaptations, including ways we could improve delivery via an online platform (5 minutes).**2a. Worksheet:*

2b. Technology adaptation: A chatbot on Slack/ text messaging that regularly asks you to enter activity, feeling, and intensity of the feeling. Here is a prototype of how that might look:

2C. Technology adaptation: We were thinking of sending reminders 3-4x/day from 9am-9pm - this would be on one weekday and one weekend day where you could fill out a survey like this one.https://www.surveygizmo.com/s3/4866621/Clinician-ARC-Behavioral-Activation-Session-1-4

Week 7

***[ACTIVITY: Introducing SMART Goals]*:** Hi @channel! Thank you for your feedback last week. We are now going to introduce more of the BA treatment for you to explore and provide feedback on. There are *two parts* for this activity!*Part 1: Upward and Downward Spiral:* An important part of BA is understanding connections among situations, feelings, and actions in order to identify what contributes to *upward* (improvements) and *downward* (worsening) spirals in mood. Please see below for how we currently teach “Upward and Downward Spirals” in mood and a potential technology adaptation:*B1. Worksheet:* Here is an explanation of what an upward and downward spiral means and the current paper worksheet used to explain the concept. *Please read over and let us know in comments if you have questions and/or need any additional details to help understand upward and downward spirals.*:

B2. Technology adaptation: We think of delivering it as a diary in survey format that you could fill out 2-3 days of the week. Please try it out by filling out this survey, and *provide feedback on your experience in understanding and filling out the survey in comments*. Also add any difficulties in understanding the content or additional details you may need to help with this part of the activity: https://www.surveygizmo.com/s3/4861333/Behavioral-Activation

Part 2: Setting SMART goals: A key component of BA is helping teens to set *SMART goals and mini-steps related to making a behavioral change to create upward spirals and prevent downward spirals in mood.* SMART stands for Specific, Measureable, Appealing, Realistic, and Time-bound goals. Please see our explanation on how to set SMART goals in the PDF, B1. In part B2, try out an interactive way of using peer to peer direct message to help each other set SMART goals (15 min)*B1. Worksheet for guidance:*

B2. Technology Adaptation: We will pair you up in a direct message channel where we'd like you to help each other set a SMART goal and reminders within Slack to work through your mini-steps. Please guide each other through the following prompts:(*a*) What's one SMART goal that you can work towards in the next week? Bonus points if it's mood boosting! (each share your SMART goal over direct message)(*b*) Respond to your partner with your thoughts on how "SMART" you think their goal is:- Is it Specific? Yes/No (any explanation)- Is it Measurable? Yes/No (any explanation)- Is it Appealing? Yes/No (any explanation)- Is it Realistic? Yes/No (any explanation)- Is it Time-bound? Yes/No (any explanation)- Walk through the mini-steps of how you are going to attain your SMART goal- as few or as many as you need. Help each other determine whether you are truly breaking things down into small, manageable steps.- Set a date/time that you'd like to accomplish each step, share it with your partner, and set reminders on your calendar, phone, or Slack (by messaging the "/remind" command on Slack message box e.g. /remind me to drink Kambucha at 5 pm on March 12)(*c*) Once you have had the chance to try direct messaging, please *give us feedback* on the Group Channel for what went well and what was challenging with this format. Feel free to share progress on your SMART goal mini-steps so we can cheer you on! There might be challenges in coordinating with each other, let us know if you encounter any difficulties. (edited)Please let me know if you have any questions, thank you so much! :

Week 8

[ACTIVITY: OVERCOMING BARRIERS TO SMART GOALS]: Hi @channel! Now that you all have had a chance to practice setting SMART Goals by direct message (and maybe completing a few mini-steps yourself!), the next take-home activity in BA involves overcoming barriers to mini-steps and SMART Goal completion.

There are two key types of barriers:*Internal Barriers:* Barriers that arise from within, such as "not feeling like it"/low motivation, feeling overwhelmed, trouble communicating needs, forgetting, or distraction.*External Barriers:* Barriers that arise outside of yourself, such as not having the needed "tools" (e.g, school book, art supplies, homework assignment etc), needing help from others (e.g., ride from parents, quiet space in the home, tutor/homework help), other people not following through on plans, or needing money.

We have several ideas for how we can build in a "barriers assessment" into our SMART goal setting and planning and would like your feedback on.

A1. Worksheet on overcoming barriers to SMART Goals: Please *read through it for reference and post feedback and ideas* on how you would like it to be implemented on technology platforms.

A2. Get to know your SMART Goal buddy: We thought it would be helpful to get to know your SMART Goal partner a bit more, please share what one thing on your bucket list is and why in your direct messages!

A3. Technology Adaptation 1: Peer support in Direct Message (message each other): In the paired groups @rianagar created last week, check-in with your SMART Goal buddy on progress and barriers with their mini-steps. Please guide each other through the following prompts:

1. How did it go completing your mini-steps to your SMART Goal? For any mini-step *not completed*, what kind of barriers got in the way (Internal or External)? If you completed all your mini-steps, you can think of a barrier that might come up in the future that you would want to overcome and brainstorm.

2. How might you get around this barrier so you can continue working towards your SMART Goal this week?

- Recommended suggestions: remember to follow a GOAL or PLAN and not a mood (Goal-Directed Behavior); think of a "goal buddy" who can help you or who can check in with you throughout the week

3. Re-set a date/time that you'd like to accomplish each step, share it with your partner, and set reminders on your calendar, phone, or Slack (by messaging the "/remind" command on Slack message box e.g. /remind me to drink Kambucha at 5 pm on March 16)

4. Post your feedback on this group Channel for what went well and what was challenging with this format. Feel free to share progress on your SMART goal mini-steps and overcoming barriers!

Week 9

[ACTIVITY: OVERCOMING BARRIERS TO SMART GOALS Part 2]: Hi @channel! The following two technology adaptations are a continuation from last week's activity on barriers to SMART Goals. Please *try them out* and *provide us feedback*.

A3. Technology Adaptation 2: Survey (Fill out the survey) Link to a survey on which you can reflect on the barriers to your mini steps in your own time and send an email to yourself with your responses. If you completed all your mini steps to your SMART Goal, think of any barriers you may have in the future mini steps while trying out the survey. -- > https://www.surveygizmo.com/s3/4891392/week8

A4. Technology Adaptation 2: (Provide us feedback on the mock-up) A continued mock-up of the SMART Goal chat-bot from two weeks ago (attached PDF with 3 pages) that follows up on (1) whether you completed each mini-step to your SMART Goal, (2) what were the barriers, and (3) scaffold revision of mini-steps for next week. *Please provide us feedback* on the chatbot mock-up.

Let us know your feedback: what can make this more helpful (if anything) and what might be difficult.

We hope you can complete this by June 3rd. Let me know if you have any questions, thank you so much!

Week 10

[ACTIVITY: HOW TO TEACH THERAPY CONTENT ON AN ONLINE PLATFORM]: We are now going to start exploring how we can teach therapy material through Slack. In order to do this, we must explore many possible options to find the best way to deliver this material in an engaging and understandable way.

Please provide as much feedback as possible!

Teaching content format: We came up with *five* possible ways to teach therapeutic content via Slack (or another online platform). *Please review* and then *complete the tasks outlined in 1 - 3 below*:

-***Powerpoint slides with voiceover***: This approach would use powerpoint slides of the worksheets, like the ones you all reviewed throughout this study (see example below as a reminder). We would add a voiceover of an expert like a school psychologist, licensed mental health provider, psychiatrist, or clinical psychologist to walk teens through the powerpoint slide concepts each week.

- ***Animated video with voiceover**:* We have a great example of what this teaching process could look like. Please note this is an example only, and not related to BA. Watch the video for at *last 1-2 minutes* to understand the general idea: https://www.youtube.com/watch?v=C_3phB93rvI.

- ***Video of a mental health professional**:* This would involve an expert like a school psychologist, licensed mental health provider, psychiatrist, or clinical psychologist explaining the concept in-person through a video format.

- ***Video of a teen or adult with lived experience**:* This would be a video of a teen or young adult explaining a concept through the lens of having experienced depression in the past. Here is an example, not specific to BA (please note this video is long, so no need to watch the entire clip): https://www.youtube.com/watch?v=njESlZa2b10.

- ***Chatbot**:* A chatbot would interactively walk through a new concept with the teen and have the ability to ask the teen questions that could later be used when coaching through a homework assignment. Please read through the mock chatbot example attached.

***1**:* Please fill out the poll to check which options you would think would be best for teaching a teen therapy content:

/poll "Please click on all teaching methods you prefer" "Animated Video with Voiceover" "Video of a mental health professional" "Video of a teen or adult with lived experience" "Chabot"

***2**:* What other ideas do you have for teaching therapy content?

***3**:* Please tell us why you selected the options in the poll.

Please try and have this done by *Monday, June 10th*, thank you so much!

Interview Protocol for Teenagers

Time: 30- 40 min

Hi, is this Participant_name?

This is <researcher_name> from the University of Washington regarding the interview on technology and teen depression management study. Thank you for taking the time out! Is this still a good time to chat?

Information regarding the interview.

The interview will take 30-40 min. I would like to learn about treating depression and your experience in the study.

I will be asking you questions on your experience in the study and on adapting behavioral activation for online platforms, and reflection on the method of using Slack.

Share only what you are comfortable with. If you feel like you don't want to answer a question, you can feel free to let me know and we can move on. You can also let me know if you want to end the interview at any time or ask me questions anytime.

Is it okay if I audio record this interview? I will delete all identifying information (name, location) and recording will be transcribed anonymously.

Experience with depression and/or low mood

Current or past experience with depression and treatment:

- Did you seek any kind of counseling, therapy, or treatment for difficulties with mood or depression? -
    - what kind of clinic/therapist,
    - how long,
    - frequency of sessions
- If you had wanted to get treatment for mood or depression and didn't, what were your barriers to getting help?

Adapting BA to online platforms like Slack

1. BA can be delivered in a long **format** (full ~12 session treatment protocol) or a short (4-6 session protocol) format.
    - What do you think are the **benefits** of long vs the short format?

- What do you think are the **challenges** of long (full ~12 session treatment protocol) vs short (4-6 session protocol) format?

2. If we think about in-person therapy to online only therapy as a spectrum, which type of care do you think would be appropriate for **you**?

3. **Typical therapy for depression** involves weekly in-person sessions. Through this project, we are hoping to use an online platform like Slack to decrease the amount of in-person treatment sessions needed and support teens in their therapy goals between sessions. This could be especially helpful for teens that live far away from mental health care and have trouble making it in every week.

   For example, a therapist would see a teen in-person once every 2-4 weeks and then use the online platform to deliver treatment, help with homework completion, and answer questions until they are able to see them next.

   What do you think about using Slack in this way to help deliver depression treatment to teens?

4. In terms of time, how burdensome do you think the prototypes for homework activities will be for you?
   - If they have therapy experience ask how is this online format different?

5. Do you have any **concerns about privacy** when using remote technologies for treatment?
   - What steps might help mitigate these privacy concerns?
   - Are there any privacy concerns that you see as insurmountable?

6. If you were to be involved in **online group support** that supplements your face to face care, what privacy concerns would you have regarding the group format?

Take home activities between sessions

1. Now that you've experienced the Slack format, how do you think it could best support teens between sessions?

   What do you think Slack can be used for to best support teens in between therapy sessions?

2. How was your experience with **the direct messaging** (DM) activity for SMART goals (if not already covered) [probe: what worked, what did not work]?

Teaching components

1. In our next phase of the study, we hope to propose design ideas for how to teach concepts via an online platform. Based on your responses, many of you indicated that an animated video with voiceover, video of teen with lived experience, or chatbot would be the best ways to teach this content online.
    a. What are your thoughts about the benefits/challenges of these approaches in teaching BA concepts?
    b. Are their other ideas you'd like to share with us?

2. Many of you indicated chatbot was not a preference, what are other ways do you think we can support the online homework format?
    a. [in person was important, supplement the treatment]
    b. Peer teaching/mentoring with a more advanced "buddy"?

3. We are thinking of a **peer learning format** for learning and practicing skills of BA. Thinking about an online platform like Slack, how would you modify the format or the interface it to support:
    a. Peer interactions among teens
        i. How many teens per group?
        ii. Synchronous elements such as a time window or video/audio chat?
        iii. Duration of the group? We are thinking 6 weeks.
    b. Safety & Moderation: who should be moderating? Safety concerns? Any suggestions for moderation practices?

Reflect on ARC Method

1. Have you used Slack before you joined the study?
    a. How was your experience using Slack during the study?
    b. Did you have notifications on or off?
    c. Activity timing -- asynchronous nature, flexibility
    d. When did you usually do the activities once it was posted?
    e. Was 20 minutes okay for each activity?
    f. Did you read others' activities
    g. How did you feel about your interactions with others on the group? - reciprocity/acknowledgements

2. What was **helpful?**

3. What was **challenging**? Any negative experiences?

4. What would have made it an easier or better experience for you (if anything)? (probe: timing: 20 minutes per activity, 1 week, any breaks?)

Wrap up:
Do you have any questions for me or regarding the study?

Researcher: Would you like to be contacted in future to provide feedback on iterations of the designs or their ideas?

Finally-- survey on for 10-15 min.

Is it okay if we send you the compensation gift card ($15) at the end of next week after all interviews?

Thank you!

Interview Protocol for Clinicians of Teenagers

Time: 30- 40 min

Hi, is this Participant_name?

This is <researcher_name> from the University of Washington regarding the interview on technology and teen depression management study. Thank you for taking the time out! Is this still a good time to chat?

Information regarding the interview.

The interview will take 30-40 min. I would like to learn about treating depression and your experience in the study.

I will be asking you questions on your experience treating teens, adapting behavioral activation for online platforms, and reflection on the method of using Slack.

Share only what you are comfortable with. If you feel like you don't want to answer a question, you can feel free to let me know and we can move on. You can also let me know if you want to end the interview at any time or ask me questions anytime.

Is it okay if I audio record this interview? I will delete all identifying information (name, location) and recording will be transcribed anonymously.

Experience with Depression Treatment for teens

You may have already answered these questions. I am asking in case there are any updates.

1. Where (what kind of setting) do you currently work? (i.e., school, hospital, community clinic)
2. How many years have you been licensed as a mental health professional?
3. How many years have you been treating children and adolescents?
4. Of the clients you have seen, what is the % teens you've treated for depression (based on your best estimate)
5. % teen depression cases where you used Behavioral Activation (based on your best estimate) → how many years have you been using it?
6. How many times per week do you see (or try to see) a teen with depression for a therapy session?

Adapting BA to online platforms like Slack

1. BA can be delivered in a long **format** (full ~12 session treatment protocol) or a short (4-6 session protocol) format.
    - What do you think are the **benefits** of long vs the short format?
    - What do you think are the **challenges** of long (full ~12 session treatment protocol) vs short (4-6 session protocol) format?

2. **Stepped care models** typically involve first delivering a low burden intervention and monitoring response for 4-8 weeks to see whether a higher level of care, like weekly individual therapy, is necessary.

    For example, a clinician would see a patient in-person once every 2-4 weeks and primarily use the technology platform to deliver initial care before moving to a higher level care (i.e., weekly in-person, referring to speciality care).

    What do you think about using a technology platform like Slack as a low burden, first step in the context of a stepped care model?

3. Clinicians overall indicated that in-person face to face sessions are preferred over only remote therapy over an online platform.
    - If we think about in-person therapy to online only therapy as a spectrum, which type of care do you think would be appropriate for **which type of teen patient**?

4. We want to make sure a technology platform is feasible for clinicians in regards to time burden. Do you need to meet certain billing requirements as part of your work for the online work you do?

    - Do you use texting, phone calls, or other remote methods to communicate with clients during therapy?
    - **If so**, does your clinic currently have a mechanism for billing for online or telehealth?
    - Has your clinic ever attempted to find a way to bill for technology-based contact with clients, like texting, email coaching etc.?

    - **If not**, how burdensome would you see monitoring your patient's/client's activity on a technology platform compared to weekly in-person visits?

5. Through the study we learned that many of you were **concerned about privacy** when using remote technologies for treatment.
    - What steps might help mitigate these privacy concerns?
    - Are there any privacy concerns that you see as insurmountable?

Take home activities between sessions

1. Now that you've experienced the Slack format, how do you think it could best support teens between sessions?

   What do you think Slack can be used for to best support teens in between therapy sessions?

2. How was your experience with **the direct messaging** (DM) activity for SMART goals (if not already covered) [probe: what worked, what did not work]?

Teaching components

1. In our next phase of the study, we hope to propose design ideas for how to teach concepts via an online platform. Based on your responses, many of you indicated that an animated video with voiceover, video of teen with lived experience, or chatbot would be the best ways to teach this content online.
   a. What are your thoughts about the benefits/challenges of these approaches in teaching BA concepts?
   b. Are their other ideas you'd like to share with us?

Reflect on ARC Method

1. Have you used Slack before you joined the study?
   a. How was your experience using Slack during the study?
   b. Did you have notifications on or off?
   c. Activity timing -- asynchronous nature, flexibility
   d. When did you usually do the activities once it was posted?
   e. Was 20 minutes okay for each activity?
   f. Did you read others' activities
   g. How did you feel about your interactions with others on the group? - reciprocity/acknowledgements

2. What was **helpful?**

3. What was **challenging**? Any negative experiences?

4. What would have made it an easier or better experience for you (if anything)? (probe: timing: 20 minutes per activity, 1 week, any breaks?)

Wrap up:

Do you have any questions for me or regarding the study?

Researcher: Would you like to be contacted in future to provide feedback on iterations of the designs or their ideas?

Finally-- survey on for 10-15 min.

Is it okay if we send you the compensation gift card ($15) at the end of next week after all interviews?

Thank you!
