# Supplementary material for: Designing Asynchronous Remote Support for Behavioral Activation in Teenagers With Depression: Formative Study
Source: JMIR Form Res. 2021 Jul 13;5(7):e20969. doi: 10.2196/20969 (PMC8317030; doi:10.2196/20969)
Supplement: Multimedia Appendix 6 [file formative_v5i7e20969_app6.pdf]

Appendix: Code Book

The following codes were used for analyzing qualitative data from interviews and online posts of both teen and clinician ARC studies. Codes were not mutually exclusive and excerpts of data had multiple codes.

Code	Description
**Access**	
Access issue face to face	Concerns about how accessing treatment face to face (f2f) is difficult due to burden on time, transport, parental support, availability of clinic, and stigma
Access issue online	Concerns that teenagers would be unable to have access to the resources appropriate for online treatment
Access benefit online	Participants mentioning reasons for patients to have better access to therapy online
**Client-therapist relationship**	
Better rapport with clinicians	Signifies the perceived need for building rapport and have human connection with clinicians, which participants perceived was more cohesive in face to face interactions than online interactions
Online boundaries	Clinicians express concerns and ideas about time spent online and setting expectations with clients on when to expect responses on a platform that is available asynchronously and 24x7. These included concerns of clinicians about being contacted outside hours working
Patient accountability	How to hold teens accountable for doing the assignments required?
Teen engagement	Perceived concerns and discussions about the engagement (and lack of engagement) of teens in the online treatment format
Parental Involvement	Teens mention about the need for parent involvement including transportation and payment; also includes where they mention parental involvement may not be

	necessary; clinician discussions on parental involvement
**Added burden of online work**	
Billing concerns	Any billing or payment related discussions for remote treatment; for example, therapy delivered online or the time spent online by clinicians is not billable currently in their practices
Clinician burden online	Lack of information that exists for clinicians when it comes to online platforms such as Slack. Added time burden for moderating and responding to posts.
Liability concern	Will clinicians be liable for anything they are unable to address online, especially, adverse events.
**Online Platform**	
Privacy concerns	Concerns expressed by clinicians about privacy and HIPAA compliance on Slack; concerns of teens about anonymity, online disclosures, and data sharing policy of Slack
Teen communication styles	Addresses concerns about how teens communicate, what their usual practices are in terms of how they communicate with others around them and what would be the most beneficial circumstances for them in regard to communication, essentially leading to what they might need online when communicating with a possible therapist and/or group of patients
Platform interface difficulty	The difficulties that clinicians and teens express with Slack specifically with regards to the interface
Platform adoption concern	The concerns clinicians share about adopting Slack into their current workspaces. These adoption concerns included the policy and security in their workplace with using an external tool such as Slack, their computer set up, or their personal ability to use technology such as this platform
Content organization on the platform	The organization of content on Slack chats and different channels.

Platform notification	whether notifications tend to be turned on or not; why or why not
Instructions for using platform	Need for adding instructions on how to use a platform that was unfamiliar to some participants.
**Technology based functionalities**	
Chatbot	Using automated prompts for interacting with teen patients
Online reminder	Feature to remind patients to do homework or do BA assignments
In-the-moment support for crisis	Support that would be necessary in an emergency with a patient
Online possibilities	Other functionalities that can be added on online platforms for benefiting teens
**BA Format**	
Long format benefit	This includes the benefits of the longer format of BA therapy (i.e.12 weeks)
Long format challenge	This includes the challenges mentioned with the long format of BA therapy
Short format benefit	This includes the benefits of the shorter format of BA (i.e. 4-6 weeks)
Short format challenge	This includes challenges mentioned for the shorter format of BA
Online check-in support	Slack being used as a support tool when patients need additional non-therapeutic assistance such as sending reminders, check-ins with clinicians
Supplement online therapy	Combination of both online therapy and in person therapy by delivering and teaching therapeutic content online

One on one online therapy<br><br>Online_group_therapy<br><br>Online_therapy_ideas	comments about the need for one on one therapy online rather than group therapy<br><br>possible group therapy online<br><br>Other comments about the benefits and ideas related to therapy online
**BA therapy critique**	Critique of the content of BA therapy (and not the technical adaptation). An example included critiquing the language that can be perceived to set expectations of being always positive.
**Teaching**<br>Teaching techniques<br><br>Interactive components	 Some techniques mentioned on how to remotely teach BA to teens<br><br>How to make the content more engaging in order to ensure teens are doing the work. For example, comments about how the animated video is good; concern expressed about video ideas presented.
**Clinician experience**<br>Previous BA therapy experience<br><br>Patient meeting frequency	 Have the clinicians used BA with their patients in the past and how often<br><br>How often are clinicians meeting their patients or teens seeing their therapists
**ARC study design**	Comments on how to change the study or add ideas, or appreciation for current study design (e.g., amount of time spent on activities, asynchronous nature of activities, and content)
